# Metabolic flux profiling of MDCK cells during growth and canine adenovirus vector production

**DOI:** 10.1038/srep23529

**Published:** 2016-03-23

**Authors:** Nuno Carinhas, Daniel A. M. Pais, Alexey Koshkin, Paulo Fernandes, Ana S. Coroadinha, Manuel J. T. Carrondo, Paula M. Alves, Ana P. Teixeira

**Affiliations:** 1 IBET, Instituto de Biologia Experimental e Tecnológica, Apartado 12, 2780-901 Oeiras, Portugal; 2 Instituto de Tecnologia Química e Biológica António Xavier, Universidade Nova de Lisboa, Avenida da República, 2780-157 Oeiras, Portugal

## Abstract

Canine adenovirus vector type 2 (CAV2) represents an alternative to human adenovirus vectors for certain gene therapy applications, particularly neurodegenerative diseases. However, more efficient production processes, assisted by a greater understanding of the effect of infection on producer cells, are required. Combining [1,2-^13^C]glucose and [U-^13^C]glutamine, we apply for the first time ^13^C-Metabolic flux analysis (^13^C-MFA) to study E1-transformed Madin-Darby Canine Kidney (MDCK) cells metabolism during growth and CAV2 production. MDCK cells displayed a marked glycolytic and ammoniagenic metabolism, and ^13^C data revealed a large fraction of glutamine-derived labelling in TCA cycle intermediates, emphasizing the role of glutamine anaplerosis. ^13^C-MFA demonstrated the importance of pyruvate cycling in balancing glycolytic and TCA cycle activities, as well as occurrence of reductive alphaketoglutarate (AKG) carboxylation. By turn, CAV2 infection significantly upregulated fluxes through most central metabolism, including glycolysis, pentose-phosphate pathway, glutamine anaplerosis and, more prominently, reductive AKG carboxylation and cytosolic acetyl-coenzyme A formation, suggestive of increased lipogenesis. Based on these results, we suggest culture supplementation strategies to stimulate nucleic acid and lipid biosynthesis for improved canine adenoviral vector production.

Adenovirus vectors have attracted considerable interest as vaccines^1^ and gene delivery systems for clinical applications^2^, including cancer therapy^3^. Inherent advantages of these vectors are the high transduction efficiencies of a broad range of cell types, their large capacity for foreign DNA accommodation and ease of *in vitro* propagation. Although a substantial fraction of the human population produces neutralizing antibodies against human adenovirus type 5 (HAV5)^4^, it has been one of the most exploited vectors used in gene transfer studies, and has prompted optimization efforts on HAV5 production from cultured host cells, including the design of nutrient feeding strategies to overcome depletion of essential nutrients and limit the build-up of toxic by-products^5^,^6^. The pre-existing immunity against HAV5 has nevertheless hampered consistency of gene transfer and encouraged the development of vectors from nonhuman origin and alternative serotypes, such as the canine adenoviral vector type 2 (CAV2)^7^. This vector has shown an extraordinary potential to transduce neurons, thus making it a good candidate for the treatment of neurodegenerative diseases^8^,^9^. CAV2 have been produced in the DK (dog kidney) cell line^10^ and more recently in the MDCK (Madin-Darby Canine Kidney) cell line^11^,^12^. However, in order to assure sufficient supply for clinical trials, production processes that facilitate the generation of large volumes of high-titer, clinical-grade vector stocks are needed. For this, it is essential to have a deeper understanding of the complex molecular interaction through which the virus takes advantage of cellular physiology to optimize its own replication.

Studies of host-virus interactions have reemphasized the critical role of cell metabolism in enabling the production of viral particles^13^,^14^,^15^. This is expected since viral synthesis requires a significant commitment of host resources, including precursors of nucleic acids, proteins and energy. For instance, microarray analysis of host cell gene expression during adenovirus infection showed that nearly 66% of the up-regulated genes encoded proteins involved in cell metabolism^16^,^17^. In another example, Munger and coworkers^14^ demonstrated that infection of human fibroblasts with cytomegalovirus (HCMV), also a DNA virus, markedly disrupts metabolic homeostasis, producing metabolome alterations in pathways such as glycolysis, the TCA cycle, and pyrimidine biosynthesis. Fluxome alterations probed through ^13^C-labelling showed HCMV upregulated the flux through much of the central carbon metabolism. In agreement, recent findings show that human adenovirus activates MYC to promote elevated expression of specific glycolytic enzymes^18^ as well as glutamine transporters and glutamine catabolism enzymes^19^ to upregulate the utilization of these substrates. However, metabolic alterations at the metabolome and fluxome levels induced by adenovirus infection of producer cell lines are not well described so far. Previous studies on adenovirus vector production were based on classical metabolic flux analysis (MFA), relying solely on metabolite balancing and measurements of extracellular transport fluxes^20^,^21^,^22^. Although providing a global snapshot of metabolism, this technique is unable to provide sufficient resolution of intracellular fluxes, namely the discrimination of parallel pathways or reversible reactions^23^. The same MFA technique has been used to study MDCK cells metabolism in microcarrier cultures^24^, and a global metabolomics profiling of this cell line was undertaken to evaluate the effect of influenza virus infection^25^.

Over the past two decades, the wealth of information arising from isotope labelling studies has made possible accurate resolution of metabolic fluxes through ^13^C-MFA^23^,^26^. Mass spectrometry (MS) or nuclear magnetic resonance (NMR) techniques are used to measure the incorporation of a ^13^C label from medium nutrients into intracellular metabolites. A computational framework based on both metabolite and mass isotopomer balances is then used to integrate the data with experimentally determined uptake and production rates to estimate metabolic fluxes. Although being much more experimentally and computationally demanding, this technique provides a level of resolution not possible with only metabolite balancing, thus leading to a clearer understanding of metabolic network operation and diagnosing of phenotypic perturbations^14^,^27^,^28^,^29^.

In the present study, we aimed at a quantitative analysis of metabolic fluxes in E1-transformed MDCK cells growing as adherent cultures and after infection by CAV2. In order to obtain a comprehensive estimation of fluxes, we performed experiments with two labelled substrates, [1,2-^13^C]glucose and [U-^13^C]glutamine, and profiled the dynamics of label incorporation in intracellular metabolites during the initial 24 h through GC-MS. Nonstationary ^13^C-MFA^30^,^31^ was applied to integrate dynamic mass isotopomer data from both tracers with extracellular uptake and production rates under growth and infection conditions. We show that the metabolism in MDCK cells is characterized by a highly glycolytic metabolism and excretion of pyruvate-derived by-products in excess of glucose consumption, while the TCA cycle is significantly fueled by glutamine anaplerosis, entailing a particular wiring of fluxes at the level of pyruvate cycling. Moreover, these cells displayed reductive AKG carboxylation activity under normal conditions. Upon CAV2 infection, our data reveals a significant upregulation of overall metabolic fluxes, including glycolysis, pentose-phosphate pathway (PPP), glutamine anaplerosis and, even more markedly, reductive AKG carboxylation and cytosolic acetyl-coenzyme A formation, likely linked with increased lipogenesis. These results are discussed in the context of other viral infections and potential strategies for bioprocess optimization of CAV2 production are suggested.

## Results

**Figure 1:**
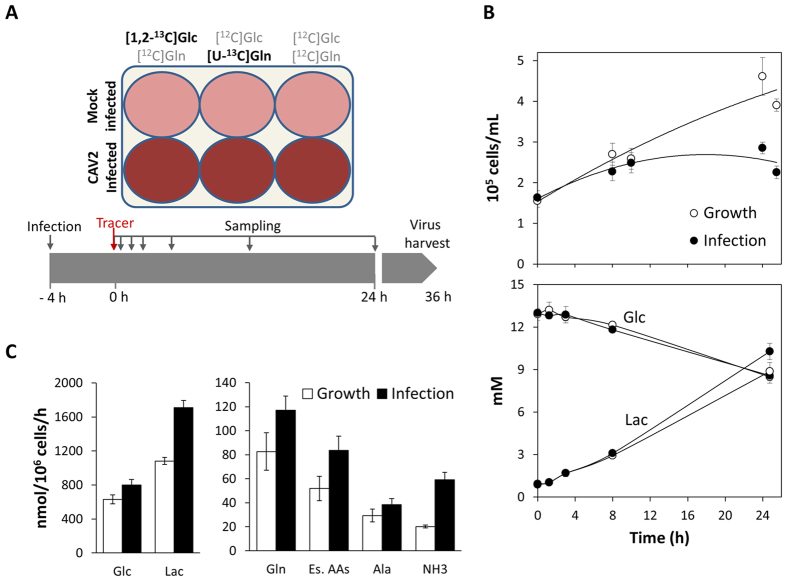
(**A**) Schematic representation of the experimental design consisting of parallel cultures of mock-infected and CAV2-infected MDCK cells incubated with [1,2-^13^C]glucose and [U-^13^C]glutamine, as indicated. (**B**) Cell growth, glucose and lactate profiles. The horizontal axis represents time after label addition in both conditions. Label was administered after 4 h of PBS or virus addition. Each time point corresponds to an average of independent replicate wells, and different time points correspond to different wells. (**C**) Exchange rates of main nutrients and by-products. Values represent average rates and associated standard deviations determined during the first 24 h after label administration. Es. AAs refers to the cumulative uptake of all essential amino acids measured.

To gain a more detailed understanding of MDCK cells metabolism and the impact of CAV2 production, we performed carbon labelling experiments combining two substrates, [1,2-^13^C]Glucose and [U-^13^C]Glutamine, which were administered in parallel cultures of both mock-infected and CAV2-infected cells (Fig. 1A). The combination of these two tracers provides an optimal setting to investigate central carbon metabolism in mammalian cells: [1,2-^13^C]Glucose provides the most information on glycolysis and pentose-phosphate pathways, while [U-^13^C]Glutamine allows the greatest resolution of TCA cycle and related fluxes^32^. This information was combined with a comprehensive analysis of culture behaviour, including extracellular metabolite consumption/production, cell growth and viral vector production.

### Culture behavior during cell growth and infection

The increase in viable cell density over time was significantly diminished after viral vector inoculation, indicating an effective infection (Fig. 1B). The specific CAV2 yield analyzed 40 h post-infection was 1835 ± 255 IP/cell, representing an amplification factor of around 245.

MDCK cells metabolism was characterized by a high rate of glucose consumption, which was mostly diverted to lactate formation (Lac/Glc = 1.71) (Fig. 1B,C). A high glycolytic metabolism has been reported before in these cells^33^. These rates were even higher for infected cells. Interestingly, lactate production increased substantially more when compared to the increase in glucose consumption, resulting in a Lac/Glc ratio that stood over 2, implying a need for additional carbon sources. Correspondingly, not only glutamine, but all of the consumed amino acids, were taken up at higher rates during infection (Fig. 1C and Table S1). To compensate the excess nitrogen uptake, the production rates of glutamate, alanine and particularly ammonia were significantly increased.

### Labeling patterns produced from [1,2-^13^C]glucose

**Figure 2:**
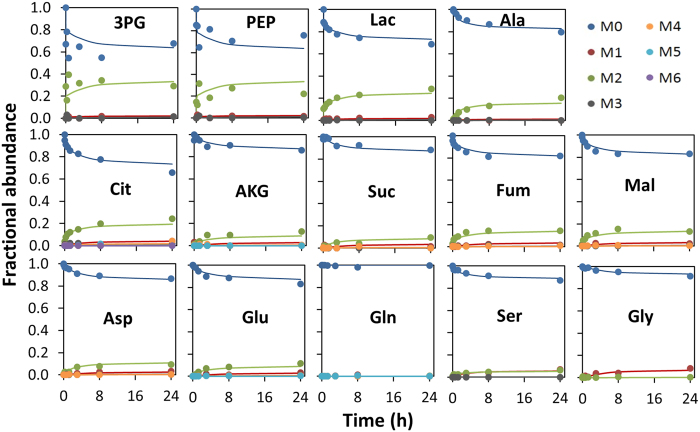
Intracellular ^13^C-labelling dynamics from [1,2-^13^C]glucose during growth (mock infection). Upon label administration, the fraction of [1,2-^13^C]glucose measured in the supernatant was 73%. Symbols correspond to GC-MS measurements corrected for natural isotope abundance. Lines correspond to fitted MIDs from nonstationary ^13^C-MFA flux estimation of parallel labelling experiments.

**Figure 3:**
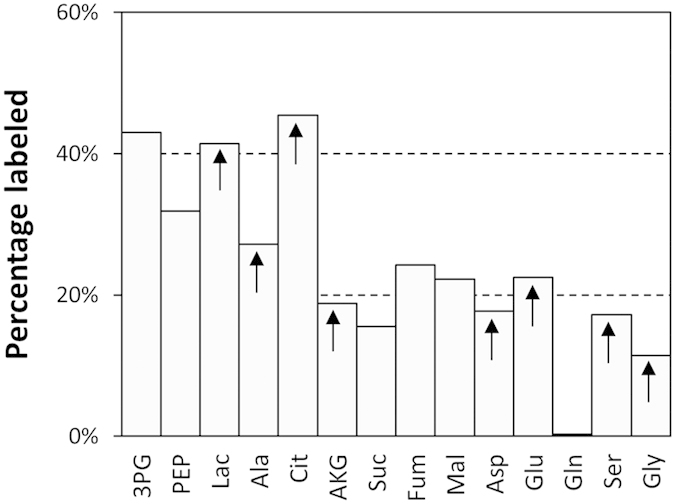
Percentage of labeled mass isotopomers (100%-M0) in intracellular metabolites from [1,2-^13^C]glucose, measured at 24 h during growth (mock infection) conditions. Values were corrected for natural isotope abundance and normalized by the corresponding percentage labeling in extracellular glucose. Arrows indicate that isotopic steady-state has not been reached.

Mass isotopomer distribution (MID) profiles of intracellular metabolites from mock-infected cells after [1,2-^13^C]glucose administration are shown in Fig. 2. Overall, the labeling patterns from CAV2-infected cells were similar (Fig. S1), indicating that essentially the same metabolic routes are operating after CAV2 infection, although quantitative flux differences exist (see below). Glycolysis and TCA cycle metabolites reached isotopic steady-state within the first 24 h, with the clear exception of citrate and likely AKG, whereas the profiles of amino acids and lactate show a continued increase of label enrichment. Moreover, a faster enrichment was observed for glycolytic intermediates over those in the TCA cycle, which partly results from the higher metabolic rate of glycolysis as well as the occurrence of equilibration of TCA cycle intermediates with amino acids pools. With the exception of citrate, the total percentage of labeled mass isotopomers in TCA cycle intermediates at 24 h was significantly smaller than in glycolytic intermediates, lactate or alanine (Fig. 3). The large discrepancy between citrate and the other carboxylic acids reflects the contribution of amino acids carbon to the TCA cycle, mainly glutamine (see below), and also suggests that some label is lost at the citrate node toward cytosolic acetyl-coenzyme A (AcCoA) synthesis^14^. The only metabolite analyzed that did not show any measurable enrichment was glutamine, consistent with inactivity of glutamine synthetase. Interestingly, it has been reported that glutamine-deprived MDCK cultures could grow and produce an influenza virus strain by addition of pyruvate as a non-ammoniagenic substitute, which indicates that glutamine synthesis is conditional to the nutritional environment in these cells^34^.

The MID profile of lactate shows a 3% enrichment of M1 at 24 h, which results from the cycling of G6P through the PPP and back to glycolysis as F6P and GAP (Fig. 2). M1 isotopomers of the same approximate magnitude were also observed within the analysis period in the lactate precursors PEP and 3PG, as well as in alanine (Tables S2 and S4). The labelled atom in these M1 isotopomers corresponds to the second carbon in glucose, while the first carbon is lost as CO_2_ in the oxidative branch of the PPP. This cycling activity is important to recover glycolytic intermediates when the need for reducing power (NADPH) exceeds the need for pentose precursors for nucleotide synthesis. M2 isotopomers can also be generated from continued flow through the PPP, but are primarily derived directly from glycolysis, while M3 was not observed.

Another necessity for nucleotide synthesis is the formation of one carbon (C1) units from serine’s decarboxylation to glycine. As observed in this study (Table S1) and other reports^35^,^36^, mammalian cells in culture usually take up serine from the medium and secrete glycine as part of C1 units metabolism. ^13^C data collected here further show that glucose is also utilized as a source of C1 units in MDCK cells by converting 3PG to serine. Specifically, we detected fractions of M1 (7%) and M2 (6%) serine isotopomers, and M1 (8%) glycine (Fig. 2), which translate into over 20% of these amino acids carbon being derived from glucose (Fig. 3; similar for infected cells). The absence of glycine M2 is caused by the fact that the lost carbon during serine conversion (third position) corresponds to the first labelled carbon in glucose.

### Labeling patterns produced from [U-^13^C]glutamine

**Figure 4:**
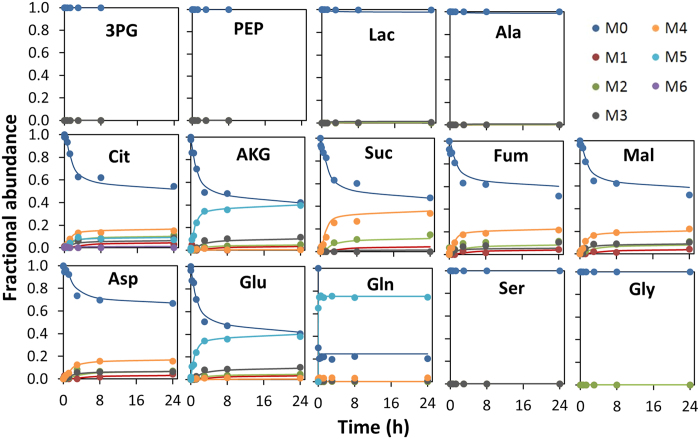
Intracellular ^13^C-labelling dynamics from [U-^13^C]glutamine during growth (mock infection). Upon label administration, the [U-^13^C]glutamine enrichment measured in the supernatant was 75%. Symbols correspond to GC-MS measurements corrected for natural isotope abundance. Lines correspond to fitted MIDs from nonstationary ^13^C-MFA flux estimation of parallel labelling experiments.

As opposed to the labelling dynamics from the glucose tracer, isotopic steady-state was not reached within 24 h of [U-^13^C]glutamine addition in all labelled metabolites (Fig. 4; similar data for infected cultures can be seen in Fig. S2). The absence of ^13^C-labelled glycolytic intermediates as well as serine and glycine indicates inactivity of gluconeogenesis, both in growth and infected conditions. Intriguingly, the absence of discernable percentages of labelled lactate or alanine would suggest inactivity of malic enzyme (ME). However, the high consumption of glutamine and the high Lac/Glc ratio suggest ME is active, particularly in the case of infected cells where an excess lactate secretion rate above the maximum allowed glycolytic rate was determined.

We could measure M3 isotopomers in Fum, Mal and Asp, but not Suc. In the TCA cycle, the generation of M3 isotopomers of these metabolites indicates activity of one or both of the following pathways: i) pyruvate cycling through ME and pyruvate carboxylase (PC); ii) reductive carboxylation of AKG to citrate via isocitrate dehydrogenase (IDH) and aconitase, followed by transport of citrate to the cytosol and ATP-citrate lyase (ACL) activity, generating cytosolic oxaloacetate (OAA) and AcCoA. The fact that Suc M3 was not discernable indicates succinate dehydrogenase (SucD) has limited reverse activity in these culture conditions. Other authors have observed reduced Suc M3 when compared to Fum and Mal in CHO cells^29^.

The inspection of the MID of citrate can provide important information on the activity of the above-mentioned pathways. We could detect a 7% fraction of citrate M5 in growth conditions (8% in infected cells), which indicates occurrence of reductive AKG carboxylation in these cells. This is corroborated by the negligible presence of citrate M6, since the M5 isotopomer can also be produced by condensing OAA M3 with AcCoA M2, while fully labeled citrate is formed in the same way but using OAA M4. Reductive carboxylation of glutamine-derived AKG has been shown to be particularly important for fatty acid biosynthesis under hypoxic conditions, for example in certain tumor environments, where metabolism is characterized by a highly glycolytic phenotype similar to that observed in this study^28^. On the other hand, the lack of a discernable fraction of citrate M6 is likely due to an extremely large glycolytic rate compared to the anaplerotic flow of carbon from glutamine consumption, resulting in the prevalence of unlabeled pyruvate. Such scenario is also consistent with the observed lactate and alanine profiles.

**Figure 5:**
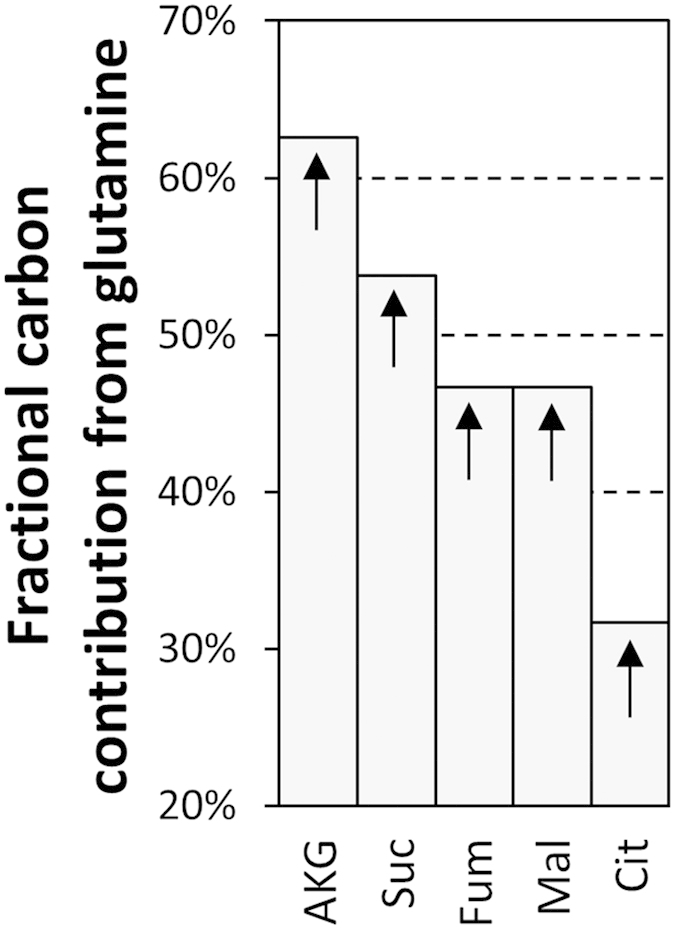
Fractional contribution of glutamine carbon to TCA cycle intermediates, calculated from the corrected mass isotopomer distributions at 24 h during growth (mock infection) conditions (sum_i_(Mi × i)/number of carbon atoms in metabolite). Values were normalized by the ^13^C enrichment in extracellular glutamine using the same equation. Arrows indicate that isotopic steady-state has not been reached.

Since we have used fully labeled glutamine as the tracer, it was possible to accurately compute the fractional contribution of glutamine carbon to TCA cycle intermediates (Fig. 5). Citrate has the lowest fractional contribution from glutamine, standing below 33% for both mock and viral vector infection. This points to a significantly greater contribution of glucose carbon to this metabolite, corroborating the high percentage of labeled citrate isotopomers from glucose shown above. Moreover, it can be seen that the fractional contribution of glutamine carbon decreases from its entry node at the TCA cycle, from AKG to citrate.

### Metabolic flux profiling during cell growth and infection

^13^C-MFA was performed based on combined nonstationary MID measurements from [1,2-^13^C]Glucose and [U-^13^C]Glutamine parallel experiments, and independently for growth and infected cultures. MID measurement errors were estimated as described in supplementary information, and the complete dataset is given in Tables S2–S5. In each flux estimation, average values of extracellular transport rates from both tracer studies were used (Table S1). The glucose and glutamine uptake rates were set fixed in order to constrain flux estimations to these measured values. Intracellular metabolite pools were not experimentally measured and were estimated by the model along the fluxes^37^. After flux estimation, acceptable fittings were obtained for all mass isotopomer fractions (Figs 2 and 4).

**Figure 6:**
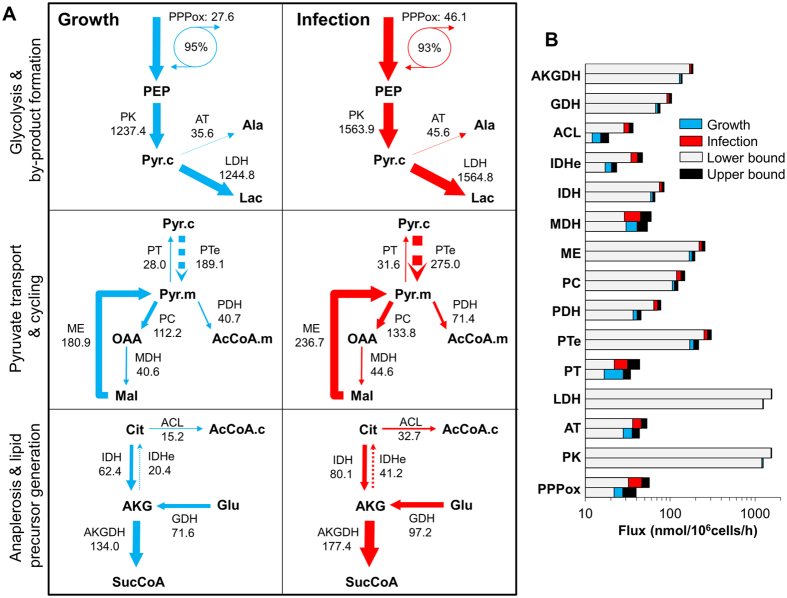
Overview of metabolic flux distributions during growth (mock infection) and CAV2 infection. (**A**) Schematic representation of metabolic flux distributions in selected reactions. Inside each pair of growth/infected panels, arrow thickness represents the relative net (full) and exchange (dashed) fluxes between conditions. Absolute values are shown in units of nmol/10^6^ cells/h. Percentage values represent the proportion of pentose-phosphates that are cycle back to glycolysis. (**B**) Estimated fluxes and associated 95% confidence lower and upper bounds of the shown reactions. ACL, ATP-citrate lyase; AKGDH, alphaketoglutarate dehydrogenase; AT, alanine aminotransferase; IDH, isocitrate dehydrogenase (lumps aconitase activity); IDHe, IDH exchange flux; GDH, glutamate dehydrogenase; LDH, lactate dehydrogenase; MDH, malate dehydrogenase; ME, malic enzyme; PC, pyruvate carboxylase; PDH, pyruvate dehydrogenase; PK, pyruvate kinase; PPPox, pentose-phosphate pathway, oxidative branch; PT, pyruvate transport flux; PTe, PT exchange flux.

Most net fluxes in the network and one third of exchange fluxes have 95% confidence intervals with finite lower and upper bounds that exclude the value zero (Table S6). Other authors have previously reported the inability to confidently estimate the majority of exchange fluxes in central metabolism^37^,^38^. A detailed representation of selected fluxes at three key metabolic branch-points and associated confidence intervals is presented in Fig. 6. As shown, a low portion of the glycolytic flux was channeled to pentose-phosphates during growth and infection, and most of it was recycled back as glycolytic intermediates. The model-estimated diversion of glycolytic flux to lactate production was approximately 100% in both conditions. The extra pyruvate needed to generate alanine for nitrogen detoxification was provided by ME activity, which resulted in a net transport of pyruvate from mitochondria into the cytosol. Consistent with the negligible presence of citrate M6 referred above, the model estimated a large pyruvate exchange flux between these compartments. This implies that both pyruvate pools are at near equilibrium which allows dilution of glutamine-derived label by the much larger glycolytic flux. In both growth and infection conditions, the flux through ME was larger than that of PC which by turn was larger than that of pyruvate dehydrogenase (PDH). Moreover, a net flux of malate formation from OAA was estimated. This pyruvate cycling activity is required to balance the highly glycolytic flow with anaplerotic incorporation of amino acids into the TCA cycle, mainly glutaminolysis where AKG is generated. AKG was then both channeled along the TCA cycle as well as converted to citrate through IDH and aconitase activities. The first of these two enzymes has not traditionally been considered to possess reversible activity, but was recently shown to be associated with the transport of citrate to the cytosol and generation of AcCoA precursors for lipid biosynthesis in certain phenotypes^28^. Of notice, the two exchange fluxes displayed were among those that could be confidently estimated due to their importance in explaining the data. Another such exchange flux was the reversed activity of SucD (Fum → Suc) which was required to be limited due to negligible Suc M3 (see Table S6).

Based on the majority set of confidently estimated intracellular fluxes, CAV2 upregulated cellular metabolism by 42% on average. In particular, the generation of pentose-phosphates through the oxidative branch of the PPP increased 67%, and a similar high portion of this flux was recycle back to glycolysis compared to mock-infected cells (Fig. 6). Even more prominently, metabolic fluxes that lead to citrate formation and its conversion to cytosolic AcCoA were specifically upregulated. Coming from glutamine, reductive AKG carboxylation (IDHe) was enlarged around 2-fold by CAV2, matching the increase in the ACL flux. At the same time, a higher increase in the fluxes through PDH (75%) and citrate synthase (46%; see Table S6) compared with other reactions around the mitochondrial pyruvate node indicates channeling of glucose carbon towards citrate formation. Importantly, this group of fluxes is among those that have non-overlapping 95% confidence intervals between mock and CAV2-infected cells (Fig. 6B).

## Discussion

In the present study, we performed a quantitative analysis of metabolism in E1-transformed MDCK cells growing as adherent cultures. We specifically aimed at assessing the metabolic impact of CAV2 infection, which constitutes a promising alternative to human adenoviral vectors for treating certain neurodegenerative diseases, while avoiding pre-existing immunity issues. In order to obtain a comprehensive estimation of metabolic fluxes, parallel experiments with [1,2-^13^C]glucose and [U-^13^C]glutamine were undertaken, and the dynamics of label incorporation in intracellular metabolites was profiled through GC-MS. Nonstationary ^13^C-MFA was applied to integrate dynamic mass isotopomer data from both tracers with extracellular uptake and production rates in growing (mock-infected) and CAV2-infected cells.

A propensity for extracellular accumulation of high levels of lactate and ammonia under normal culture conditions is common in mammalian cell culture, for example in CHO cells^29^,^35^, and has been noted before in MDCK cells^24^,^33^. From the collected ^13^C data and the determined flux distribution assessed here for growing MDCK cells, glucose was converted to lactate at near stoichiometric levels and the TCA cycle was significantly fueled by amino acid carbon sources, primarily glutamine, leading to release of the nitrogen by-products ammonia, alanine and glutamate. In this scenario, the high glycolytic flux assures most of the energetic requirements of the cell while the redox balance is maintained by regenerating NAD^+^ from NADH through conversion of pyruvate to lactate^39^. Moreover, a net flow of pyruvate away from the TCA cycle was necessary to provide the backbone for alanine production. In order to accommodate this flux while maintaining an operational TCA cycle, pyruvate cycling enzymes are rather active, mainly ME and PC. Since our ^13^C model does not explicitly consider nitrogen metabolism, the relative contributions of specific enzymes, such as glutaminase and glutamate dehydrogenase (GDH), to ammonia formation cannot be precisely determined. Nevertheless, the estimated glutaminase flux was on the same magnitude (slightly higher) than that of GDH in both mock- and CAV2-infected cells (see Table S6). As the majority of glutamine’s carbon backbone is used to fuel the TCA cycle, we expect approximately the same proportion of ammonia release from each reaction. An interesting finding of this study is that MDCK cells process a significant portion of glutamine-derived AKG through the reductive carboxylation route through reversed IDH activity. Such activity has been described to exclusively provide the necessary AcCoA for fatty acid biosynthesis under hypoxic conditions, or in certain tumor cell types even under normal conditions^28^.

CAV2 infection of MDCK cells increased fluxes through most central carbon metabolism, including glycolysis, glutaminolysis and uptake of all other consumed amino acids. Several reports have underscored that viral infection requires a particular metabolic environment that resembles that of cancer cells. The induction of a Warburg effect by infection has been described for different viruses^40^,^41^, including adenovirus^18^, linked with upregulation of glucose transporters and glycolytic enzymes. Other reports have demonstrated that glutamine anaplerosis is essential for viral replication^42^,^43^,^44^, as is an active lipogenesis pathway driven by citrate export to the cytosol^14^,^45^,^46^. In addition, it has been recently shown that optimal adenovirus infection is associated with increased use of glutamine in reductive carboxylation^19^. In our study, glucose and glutamine uptake rates were increased 27% and 42%, respectively, after infection, while IDHe and ACL activities increased over 100%. These differences indicate a greater degree of flux rewiring at the level of reductive carboxylation and likely lipogenesis. Moreover, PPP fluxes increased over 60%, highlighting biosynthetic needs for viral replication not only in terms of nucleic acid precursors, but also reducing equivalents in the form of NADPH (used for instance in reductive carboxylation and fatty acid synthesis). Interestingly, both nucleic acid and fatty acid biosynthesis have been used as targets to treat human infection with other viruses^14^,^47^,^48^. In an opposite direction, implementing strategies that upregulate these routes may benefit canine adenoviral vector production from host cell lines. For lipogenesis, this could potentially be achieved by medium supplementation of key biosynthetic precursors, such as AcCoA-generating carboxylic acids. Alternatively, direct supplementation of lipids cocktails, including fatty acids and cholesterol (proven effective during retroviral vector production under serum deprivation^49^), could be envisaged to complement/alleviate the increased biosynthetic activity triggered during adenoviral vector production. As for nucleic acids formation, supplementing the medium with nucleosides (shown to improve retroviral vector production^50^) could be attempted in opposition to the use of nucleoside analogues in antiviral and anticancer therapy. If necessary, cellular engineering strategies to increase the activity of key enzymes involved in both biosynthetic pathways should be explored. Aside from the considerable larger effort associated with this approach, it could potentially provide a synergistic effect with medium supplementation.

## Conclusions

Over the recent past, increased efforts on analytical and computational fronts have allowed ^13^C-based MFA to provide more comprehensive and detailed quantifications of flux distributions in cultured cells. At the same time, interest in investigating the metabolic impact of viral infection has gained traction and generated unprecedented knowledge on the molecular mechanisms used by viruses to manipulate their host’s metabolism. However, the application of ^13^C-MFA for intracellular flux estimation in this setting has been limited. In the present study, we generated information on MDCK cells metabolism during growth and CAV2 infection that would not be possible with simple material balance techniques, including the resolution of parallel pathways (such as i) the cycling of pentose-phosphates to glycolysis and ii) pyruvate cycling to balance carbon flow between glycolysis and the TCA cycle) and the discerning of reversed fluxes (such as the reductive carboxylation of glutamine-derived AKG, among others confidently estimated). Aside from a general upregulation of cellular carbon metabolism upon infection, we have uncovered specific pathways that play a prominent role for effective CAV2 replication and which could be explored as targets in future optimization of production processes of canine adenoviral vectors. These are nucleic acid biosynthesis and lipogenesis, two metabolic targets of antiviral therapy in the context of other human pathogens. Finally, given the valuable knowledge that these tools provide, we expect the application of ^13^C-MFA studies to understand viral infection both fo r antiviral research and viral vector bioprocess optimization will multiply in the future.

## Materials and Methods

### Cell lines and maintenance

The E1-transformed MDCK cell line, previously established at our lab^12^ by transforming MDCK cells (ECCAC Ref. 84121903) with the E1 region from Canine Adenovirus type 2, was cultured in Optipro serum-free medium (Gibco) containing 4 mM glutamine. Dog Kidney cells expressing the same E1 protein (DKZeo^8^) were used for viral stock preparation and titration, and cultured in Dulbecco’s Modified Eagle Medium (DMEM; Gibco) supplemented with 10% (v/v) Fetal Bovine Serum (FBS, Gibco). Both cell lines were routinely maintained in T-flasks, in a humidified incubator at 5% CO_2_ and 37 °C, and sub-cultured twice a week.

### Viral vector stock preparation and titration

CAV2 was previously generated by deletion of the E1 region and inclusion of an eGFP expression cassette in the Canine Adenovirus type 2 strain Toronto A26/61 (GenBank U77082)^10^. The viral stock was prepared by infecting DKZeo cells at 80–90% confluency in fresh medium, with a multiplicity of infection (MOI) of 5 IP/cell. 40 h after infection, cells were lysed with 0.1% (v/v) Triton X-100 (Sigma-Aldrich, Steinhein, Germany) in 10 mM Tris-HCl buffer at pH 8. Following 10 min centrifugation at 3000 × g, 4 °C, viral particles were purified by CsCl gradients^10^. A desalting step was then performed on an AKTA system using a HiPrep 26/10 Desalting Column (GE Healthcare, USA). CAV2 were eluted in PBS and 10% (v/v) glycerol (Sigma-Aldrich) was added afterwards. Aliquots were stored at −85 °C.

Quantification of IPs was performed by flow cytometry^11^. Briefly, DKZeo cells were incubated for 24 h with serial dilutions of viral vector-containing samples, followed by trypsin-harvesting and analysis in a Flow Cytometer (CyFlow Space, Partec, Germany) to determine the percentage of GFP-positive cells.

### Isotopic tracer experiments and sampling

For the parallel isotopic labelling experiments, two isotopic tracers were used: [1,2-^13^C]glucose (CLM-504-1) and [U-^13^C]glutamine (CLM-1822-H-0.25; Cambridge Isotope Laboratories, Andover, MA). The following 3 stock solutions of glucose and glutamine (200 mM and 40 mM, respectively) were prepared in PBS: i) ^13^C glucose and ^12^C glutamine, ii) ^12^C glucose and ^13^C glutamine, and iii) ^12^C glucose and ^12^C glutamine. Cells were seeded in 6-well plates at a density of 3.6 × 10^5^ cells/well in standard Optipro medium adding up to 2 mL total volume. After approximately 12 h, the medium was completely exchanged by 1.9 mL custom Optipro containing 3 mM and 0.5 mM of glucose and glutamine, respectively, and cells were infected with CAV2 at a MOI of 10 IP/cell or mock-infected (equal volume of PBS). After 4 hours, 100 μL of each ^13^C/^12^C stock solution (i.e., 10 mM glucose and 2 mM glutamine) were added to CAV2-infected and mock-infected wells, totalling the 6 wells in one plate (Fig. 1A).

In order to analyse label incorporation into intracellular metabolites, one plate was harvested immediately before label administration and then at each sampling time point: 1 min, 15 min, 30 min, 1 h, 3 h, 8 h and 24 h. Metabolic quenching and extraction was performed as described below. At given time points, culture supernatants from replicate wells of CAV2-infected and mock-infected cells were centrifuged at 200 × g for 10 min, 4 °C, to eliminate cell debris and stored at −20 °C for extracellular metabolite analysis (see below). Additional plates were set for assessing viable cell density in each condition and to determine CAV2 production at 40 h after viral vector infection.

### Viable cell density and quantification of extracellular metabolite concentrations

Cell concentration and viability were determined by cell counting in a Fuchs-Rosenthal haemacytometer (Brand, Wertheim, Germany) using trypan blue exclusion (0.1% (v/v) solution prepared in PBS).

Glucose and lactate concentrations in culture supernatants were measured in an YSI 7100 Multiparameter Bioanalytical System (YSI Life Sciences, Dayton, OH). Amino acid concentrations were analyzed by HPLC using the Waters AccQ. Tag Amino Acid Analysis Method (Waters, Milford, MA), as described elsewhere^51^. Ammonia concentration was measured using an enzymatic kit (Cat. No. AK00091, NZYTech, Portugal) based on the spectrophotometric detection of NADP^+^ formed by glutamate dehydrogenase activity.

### Extraction of intracellular metabolites

Metabolic quenching and extraction protocols were adapted from the literature^52^. After removal of the supernatant, cell monolayers were immediately washed with ice-cold 0.9% (w/v) NaCl. This saline solution was removed and the complete plate dipped in liquid N_2_. To extract intracellular metabolites from the frozen monolayers, 2 mL of 50% (v/v) ice-cold acetonitrile were added to each well. The content of each well was then transferred to a tube, snap-frozen again in liquid N_2_ and kept at −80 °C until analysis.

### Derivatization

Cell extract samples were thawed on ice and centrifuged at 15000 × g for 10 min, 4 °C. The supernatants were transferred to tubes and dried by vacuum centrifugation overnight. Label enrichment in extracellular glucose and glutamine was also assessed; in this case, culture supernatant samples were thawed at room temperature and dried by vacuum centrifugation overnight.

Derivatization was performed according to methods previously described^53^,^54^. Amino acids, lactate and TCA intermediates were derivatized to their *tert*-butyldimethylsilyl derivatives by adding 20 μL of a 10 mg/mL O-methylhydroxylamine hydrochloride (Sigma-Aldrich, DE) solution in pyridine (Sigma-Aldrich, DE). The reaction was allowed to proceed for 2 hours at 40 °C in a heating block, after which 40 μL of N-methyl-N-*tert*-butyldimethylsilyl-trifluoroacetamide (MBDSTFA, Aldrich, DE) were added. After 1 h at room temperature, the reaction vessel was left at 60 °C for an additional 1 h^53^. For phosphoenolpyruvate (PEP) and 3-phosphoglyceric acid (3PG), samples were incubated at room temperature with 20 μL BSTFA (N,O-Bis(trimethylsilyl)trifluoroacetamide (Supelco Analytical, DE), 10 μL TMCS (Chlorotrimethylsilane; Aldrich, DE) and 20 μL acetonitrile during 30 min^53^. In the case of glucose, 50 μL of a 20 mg/mL O-methylhydroxylamine hydrochloride (Sigma-Aldrich, DE) solution in pyridine (Sigma-Aldrich, DE) was added and samples were incubated for 1 h at 90 °C. Then, 100 μL propionic anhydride were added and the reaction was allowed to proceed for another 30 min. Finally, samples were vacuum dried and solubilised in 100 μL ethyl acetate^54^.

After completing the derivatization protocols, all samples were centrifuged for 1 min at 1000 g and the supernatants transferred to GC-MS glass vials.

### GC-MS Analysis

Samples were analysed in a QP2010 mass spectrometer (Shimadzu, Japan) in the EI mode (70 eV). The temperature of the ionic source and the interface connecting to the column was 250 °C. GC was performed on a HP-5 MS column (30 m, 0.25 mm i.d., composed of dimethylpolysiloxane with 5% phenyl groups, 0.25 mm film thickness; Agilent Technologies). Samples were injected in splitless or split (1:10) mode, using helium as carrier gas at an inlet pressure of 600 kPa. Automatic injections were carried out by an AOC-5000 Plus autosampler (Shimadzu, Japan) with an injection volume of 1 μL and injector temperature set at 250 °C. The protocol for each sample consisted in starting with an oven temperature of 120 °C, holding for 2 min, linearly increasing the temperature at 3 °C/min until 290 °C, and finally holding for 3 min as described before^55^. However, for glucose analysis a different method was used: starting with an oven temperature of 80 °C, holding for 2 min, linearly increasing the temperature at 15 °C/min until 280 °C and holding for 6 min^54^.

The obtained spectra were analysed and integrated using GC-MS Solution software version 2.50 SU1 (Shimadzu, Japan). Metabolites were identified by comparison with standard solutions prepared in water. MIDs were calculated after spectra integration and corrected for natural isotope abundance. All ions (M0, M1, Mn) were measured, where M is the mass/charge ratio of the unlabelled derivatized fragment and n is the number of labelled carbons.

### Metabolic network and nonstationary ^13^C-MFA

A reaction network was generated that included all major pathways of central carbon metabolism, including a lumped reaction for biomass formation. The cell biomass composition considered is a compilation from different sources^56^. A cell dry weight of 540 pg was determined, allowing to compute all metabolite coefficients included in the lumped biomass reaction. Metabolic precursor requirements for viral vector biomass production were negligible based on an analysis of CAV2 composition and viral vector production from infection cultures, and thus they were omitted. The negligible impact of viral production in material balances of producer animal cells has been discussed before^57^. Extracellular transport rates were considered reversible to allow equilibration with extracellular pools, except for essential nutrients (glucose, glutamine, essential amino acids). Metabolically derived CO_2_ was allowed to exchange freely with the extracellular compartment so that both labelled and unlabeled CO_2_ is available for use in carboxylation reactions^28^. As is common practice, cofactor balances were not considered. A list of all metabolic reactions including carbon atom transitions and a list of balanced/unbalanced metabolite pools are given as supplementary information.

Nonstationary ^13^C-MFA of parallel labelling experiments was performed using the publicly available software package INCA^58^, which automatically generates and simulates mass and material balance equations from a user-defined metabolic network structure and experimental datasets. Details on the algorithm for flux estimation have been extensively described^31^,^59^,^60^. At least 10 restarts with random initial values were performed to find a global optimum^61^. At convergence, the obtained solution was subject to a qui-square statistical test to evaluate goodness-of-fit. 95% confidence intervals of estimated fluxes were calculated through the parameter continuation method^59^.

## Additional Information

**How to cite this article**: Carinhas, N. *et al*. Metabolic flux profiling of MDCK cells during growth and canine adenovirus vector production. *Sci. Rep.* **6**, 23529; doi: 10.1038/srep23529 (2016).

## Supplementary Material

Supplementary Information
