# Supplementary material for: Metabolic flux profiling of MDCK cells during growth and canine adenovirus vector production
Source: Sci Rep. 2016 Mar 23;6:23529. doi: 10.1038/srep23529 (PMC4804208; doi:10.1038/srep23529)
Supplement: Supplementary Information [file srep23529-s1.pdf]

Metabolic flux profiling of MDCK cells during growth and canine adenovirus vector production

Nuno Carinhas[1#], Daniel AM Pais[1,2#], Alexey Koshkin[1,2], Paulo Fernandes[1,2,+], Ana S Coroadinha[1,2], Manuel JT Carrondo[1,2], Paula M Alves[1,2], Ana P Teixeira[1,2*]

[1]iBET, Instituto de Biologia Experimental e Tecnológica, Apartado 12, 2780-901 Oeiras, Portugal;

[2]Instituto de Tecnologia Química e Biológica António Xavier, Universidade Nova de Lisboa, Avenida da República, 2780-157 Oeiras, Portugal

[#]Equal contribution

+Current address: Autolus, London, UK

*Corresponding author: anat@itqb.unl.pt

Supplementary information: complete metabolic network model used for nonstationary ^{13}C-MFA.

	**Glycolysis**
R1	G6P (abcdef) ↔ F6P (abcdef)
R2	F6P (abcdef) → FBP (abcdef)
R3	FBP (abcdef) ↔ DHAP (cba) + GAP (def)
R4	DHAP (abc) ↔ GAP (abc)
R5	GAP (abc) ↔ 3PG (abc)
R6	3PG (abc) ↔ PEP (abc)
R7	PEP (abc) → Pyr.c (abc)
	**Pentose-phosphate pathway**
R8	G6P (abcdef) → P5P (bcdef) + CO_2 (a)
R9	P5P (abcde) + P5P (pqrst) ↔ GAP (rst) + S7P (pqabcde)
R10	S7P (abcdefg) + GAP (xyz) ↔ E4P (defg) + F6P (abcxyz)
R11	E4P (abcd) + P5P (pqrst) ↔ GAP (rst) + F6P (pqabcd)
	**Lactate and alanine accumulation**
R12	Pyr.c (abc) ↔ Lac (abc)
R13	Pyr.c (abc) ↔ Ala (abc)
	**TCA cycle and pyruvate cycling**
R14	Pyr.m (abc) → AcCoA.m (bc) + CO_2 (a)
R15	Pyr.m (abc) + CO_2 (d) → OAA (abcd)
R16	OAA (abcd) + AcCoA.m (ef) → Cit (dcbfea)
R17	Cit (abcdef) ↔ AKG (abcde) + CO_2 (f)
R18	AKG (abcde) → SucCoA (bcde) + CO_2 (a)
R19	SucCoA (abcd) ↔ Suc (abcd)
R20	Suc (abcd) ↔ Fum (abcd)
R21	Fum (abcd) ↔ Mal (abcd)
R22	Mal (abcd) ↔ OAA (abcd)
R23	Mal (abcd) → Pyr.m (abc) + CO_2 (d)
	**Lipid precursor generation**
R24	Cit (dcbfea) → OAA (abcd) + AcCoA.c (ef)
	**Amino acids metabolism**
R25	Gln (abcde) → Glu (abcde)
R26	AKG (abcde) ↔ Glu (abcde)
R27	Asn (abcd) ↔ Asp (abcd)
R28	Asp (abcd) ↔ OAA (abcd)
R29	3PG (abc) → Ser (abc)
R30	Ser (abc) → Pyr.c (abc)
R31	Ser (abc) ↔ Gly (ab) + C1 (c)
R32	Glu (abcde) ↔ Pro (abcde)
R33	Val (abcde) + CO_2 (f) → Suc (dcef) + CO_2 (a) + CO_2 (b)

R34	Ile (abcdef) + CO_2 (g) → Suc (bcdg) + AcCoA.m (ef) + CO_2 (a)
R35	Leu (abcdef) + CO_2 (g) → AcCoA.m (bc) + AcCoA.m (de) + AcCoA.m (gf) + CO_2 (a)
R36	Thr (abcd) → AcCoA.m (cd) + Gly (ab)
R37	Phe (abcdefghi) → Tyr (abcdefghi)
R38	Tyr (abcdefghi) → Fum (defg) + AcCoA.m (bc) + AcCoA.m (hi) + CO_2 (a)
R39	Met (abcde) + Ser (fgh) + CO_2 (i) → Suc (bcdi) + Cys.snk (fgh) + CO_2 (a) + C1 (e)
R40	Lys (abcdef) → CO_2 (a) + CO_2 (f) + AcCoA.m (bc) + AcCoA.m (de)
R41	His (abcdef) → Glu (edcba) + C1 (f)
R42	Arg (abcdef) → Glu (abcde) + Urea.snk (f)
R43	Glu (abcde) + CO_2 (f) → Arg (abcdef)
	**Intracellular transport**
R44	Pyr.c (abc) ↔ Pyr.m (abc)
	**Extracellular transport**
R45	CO_2 (a) ↔ CO_2.ext (a)
R46	Glc.ext (abcdef) → G6P (abcdef)
R47	Lac (abc) ↔ Lac.ext (abc)
R48	Ala (abc) ↔ Ala.ext (abc)
R49	Gln.ext (abcde) → Gln (abcde)
R50	Glu (abcde) ↔ Glu.ext (abcde)
R51	Asp (abcd) ↔ Asp.ext (abcd)
R52	Asn (abcd) ↔ Asn.ext (abcd)
R53	Ser.ext (abc) ↔ Ser (abc)
R54	Gly (ab) ↔ Gly.ext (ab)
R55	Pro.ext (abcde) ↔ Pro (abcde)
R56	Val.ext (abcde) → Val (abcde)
R57	Ile.ext (abcdef) → Ile (abcdef)
R58	Leu.ext (abcdef) → Leu (abcdef)
R59	Thr.ext (abcd) → Thr (abcd)
R60	Phe.ext (abcdefghi) → Phe (abcdefghi)
R61	Tyr.ext (abcdefghi) → Tyr (abcdefghi)
R62	Met.ext (abcde) → Met (abcde)
R63	Lys.ext (abcdef) → Lys (abcdef)
R64	His.ext (abcdef) → His (abcdef)
R65	Arg.ext (abcdef) ↔ Arg (abcdef)
	**Biomass formation**
R66	324*Ala + 208.4*Glu + 173.9*Gln + 355.4*Gly + 287.8*Ser + 307.8*Lys + 304.6*Leu + 175*Ile + 203.6*Arg + 254.8*Asp + 208.4*Thr + 224.6*Val + 74.52*Met + 118.3*Phe + 98.28*Tyr + 77.22*His + 169*Pro + 155.5*Asn + 159.8*G6P + 125.8*P5P + 129.7*C1 + 65.88*DHAP + 1362*AcCoA.c → Biomass

Coefficients in the lumped biomass formation reaction represent the nmol content in 10^6 cells. Suffix abbreviations: mitochondrial (.m), cytosolic (.c), extracellular (.ext), sink (.snk). Sink pools were used for metabolites that could not be balanced (Cys, Urea).

Supplementary information: list of balanced and unbalanced metabolite pools.

Balanced metabolite pools: G6P, F6P, FBP, DHAP, GAP, 3PG, PEP, Pyr.c, Pyr.m, P5P, CO_2, S7P, E4P, Lac, Ala, AcCoA.m, AcCoA.c, OAA, Cit, AKG, SucCoA, Suc, Fum, Mal, Gln, Glu, Asn, Asp, Ser, Gly, C1, Pro, Val, Ile, Leu, Thr, Phe, Tyr, Met, Lys, His, Arg. Unbalanced metabolite pools: CO_2.ext, Glc.ext, Lac.ext, Ala.ext, Gln.ext, Glu.ext, Asp.ext, Asn.ext, Ser.ext, Gly.ext, Pro.ext, Val.ext, Ile.ext, Leu.ext, Thr.ext, Phe.ext, Tyr.ext, Met.ext, Lys.ext, His.ext, Arg.ext, Cys.snk, Urea.snk, Biomass.

Supplementary information: calculation of MID errors for flux estimation.

In our experimental design, each MID datapoint corresponds to an independent culture, so that the measured MID dynamic profiles incorporate both analytical and biological variability. However, individual errors for MID measurements could not be experimentally determined. Therefore, a default standard error vector was initially assumed by INCA: 0.3 mol% for mass isotopomer abundances ≤0.5 mol% with linear scaling up to 1 mol% for mass isotopomer abundances ≥25 mol%. After flux estimation, reasonably good fittings were obtained for all metabolites in each culture condition, but the computed weighted sum of squared residuals (SSR) were grossly above the expected 95% confidence interval. In an attempt to further improve fitting of the data, compartmentalization of different metabolites involved in shuttle systems between the cytosol and mitochondria (such as Mal, OAA and Cit; Ahn and Antoniewicz, 2011; 2013), and use of dilution pools previously described (such as Suc; Metallo et al., 2012) were explored. However, these modifications did not materially reduce SSR values. We then concluded the reason for the high SSR values was an inappropriate account of measurement errors, indicating that the assumed errors did not represent true experimental variability. In order to better harness this variability, we subtracted MID

fittings from MID measurements for all timepoints. The resulting values were then subject to a minimum cutoff of 0.3 mol% and were used as new error estimates replacing the initial default errors assumed by INCA. After this procedure, we were able to obtain flux estimation solutions with statistically accepted fits for both growth (810.4; 786.3-949.4; 95% conf., 866 degrees of freedom) and infected cultures (853.6; 780.6-943.2; 95% conf., 860 degrees of freedom).

Ahn WS, Antoniewicz MR. 2011. Metabolic flux analysis of CHO cells at growth and non-growth phases using isotopic tracers and mass spectrometry. Metab Eng 13:598–609.

Ahn WS, Antoniewicz MR. 2013. Parallel labeling experiments with [1,2-^{13}C]glucose and [U-^{13}C]glutamine provide new insights into CHO cell metabolism. Metab Eng 15:34–47.

Metallo CM, Gameiro PA, Bell EL, Mattaini KR, Yang J, Hiller K, Jewell CM, Johnson ZR, Irvine DJ, Guarente L, Kelleher JK, Heiden MGV, Iliopoulos O, Stephanopoulos G. 2012. Reductive glutamine metabolism by IDH1 mediates lipogenesis under hypoxia. Nature 481:380–384.

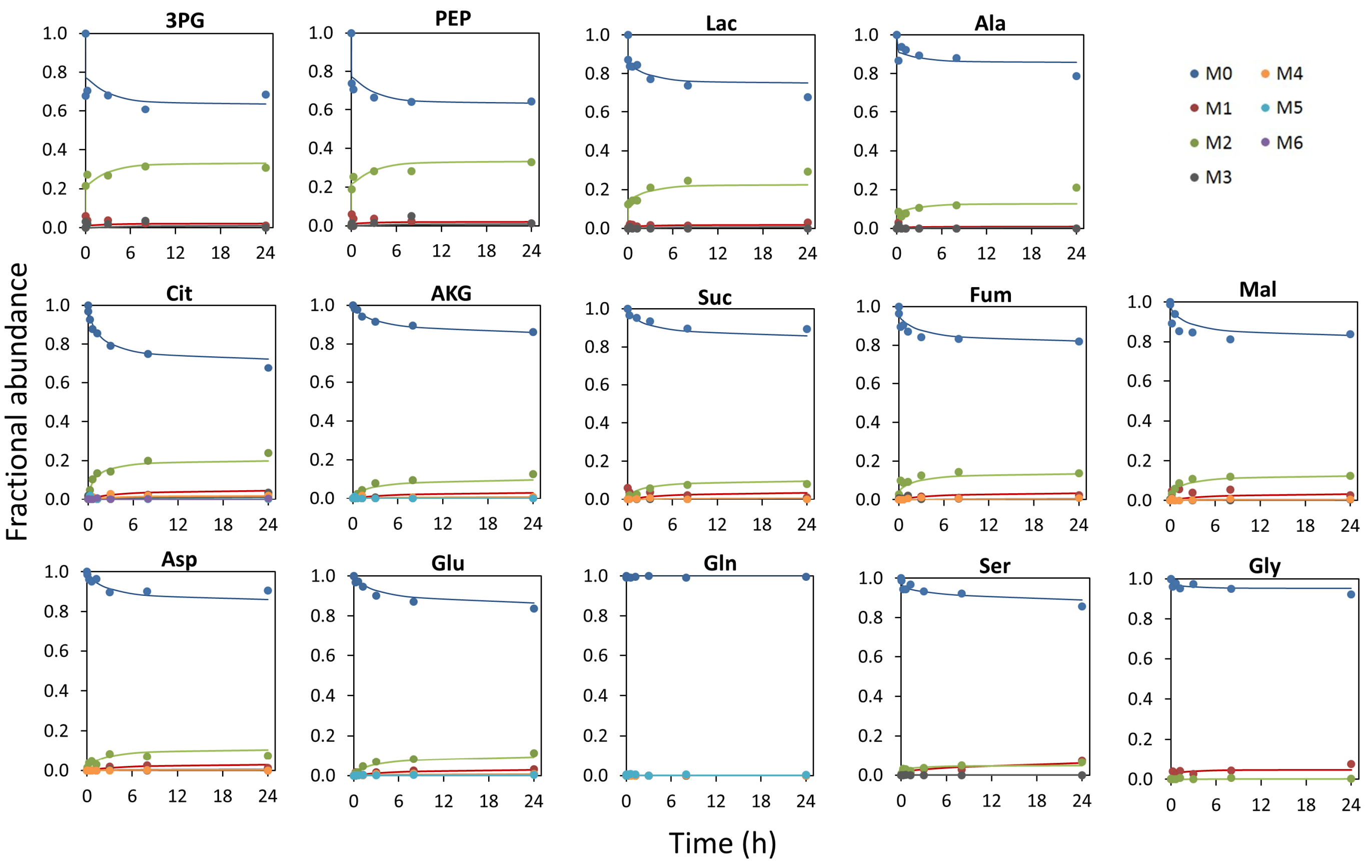

Figure S1 – Experimental and simulated intracellular ^{13}C-labelling dynamics during CAV2 infection from [1,2-^{13}C]glucose. Circle markers correspond to GC-MS measurements corrected for natural isotope abundance. Lines correspond to fitted MIDs from nonstationary ^{13}C-MFA flux estimation of parallel labelling experiments.

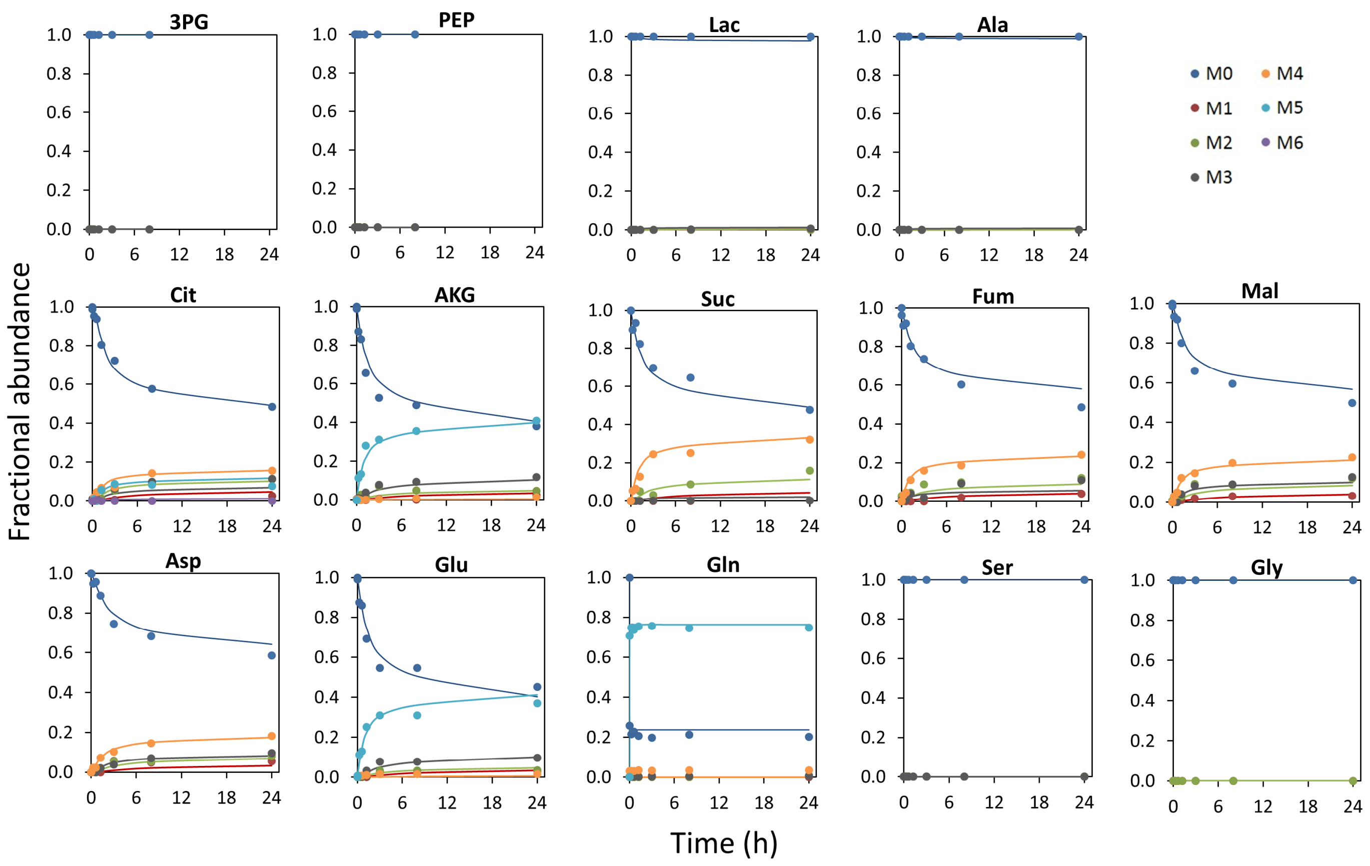

Figure S2 – Experimental and simulated intracellular ^{13}C-labelling dynamics during CAV2 infection from [U-^{13}C]glutamine. Circle markers correspond to GC-MS measurements corrected for natural isotope abundance. Lines correspond to fitted MIDs from nonstationary ^{13}C-MFA flux estimation of parallel labelling experiments.

Table S1 - Measured metabolic uptake and production rates for MDCK cells (mock-infected) and CAV2-infected MDCK cells.

	Growth	SD	Infection	SD
**Glc**	-630.77	53.47	-800.34	63.95
**Lac**	1081.95	42.05	1710.79	82.04
**NH3**	20.17	1.15	59.2	6.06
**Gln**	-82.63	15.66	-117.14	11.69
**Ala**	29.36	5.39	38.51	5.04
**Ser**	-11.89	2.49	-17.84	1.94
**Glu**	12.31	1.62	15.78	2.69
**Gly**	3.83	0.62	3.26	1.1
**Asp**	2.71	0.84	0.86	1.11
**Asn**	0.24	2.43	-6.05	4.73
**Arg**	-7.72	1.36	-12.69	0.95
**His**	-1.81	0.29	-2.57	0.44
**Thr**	-2.96	0.71	-5.67	0.96
**Pro**	-11.8	3.14	-12.77	3.66
**Tyr**	-1.64	0.25	-3.46	0.18
**Val**	-3.84	2.27	-13.71	2.2
**Met**	-4.36	0.92	-5.76	0.86
**Ile**	-7.53	0.49	-11.19	0.43
**Leu**	-10.35	0.52	-15.73	1.54
**Lys**	-5.93	1.27	-9.63	0.58
**Phe**	-1.66	0.23	-3.13	0.91
**μ**	0.0343	0.0057	0.0124	0.0072

Specifc extracellular rates were determined during the first 24 h after label administration, in units of nmol/10^6 cells/h, except biomass formation (μ = h^{-1}). Negative values indicate cellular uptake.

Table S2 - Measured mass isotopomer distributions and calculated errors from [1,2-^{13}C]glucose under growth conditions (mock infection).

**Time (h)**	**0.00**		**0.02**		**0.25**		**0.608**		**1.217**		**3**		**8**		**24**	
3PG_459 (M0)	1.0000	0.0030	0.6692	0.1231	0.7824	0.0034	0.5450	0.2313	-	-	0.6481	0.0786	0.5485	0.1242	0.6762	0.0373
3PG_460 (M1)	0.0000	0.0030	0.0440	0.0347	0.0245	0.0148	0.0600	0.0497	-	-	0.0359	0.0223	0.1095	0.0925	0.0155	0.0040
3PG_461 (M2)	0.0000	0.0030	0.2869	0.0885	0.1629	0.0414	0.3950	0.1820	-	-	0.3160	0.0582	0.3419	0.0369	0.2914	0.0406
3PG_462 (M3)	0.0000	0.0030	0.0000	0.0030	0.0302	0.0301	0.0000	0.0030	-	-	0.0000	0.0030	0.0000	0.0051	0.0169	0.0074
PEP_369 (M0)	1.0000	0.0030	0.8526	0.0042	0.8429	0.0568	0.6484	0.1281	-	-	0.8126	0.0857	0.7082	0.0354	0.7600	0.1209
PEP_370 (M1)	0.0000	0.0030	0.0000	0.0068	0.0286	0.0189	0.0173	0.0070	-	-	0.0000	0.0136	0.0179	0.0030	0.0153	0.0042
PEP_371 (M2)	0.0000	0.0030	0.1474	0.0030	0.1185	0.0854	0.3145	0.1017	-	-	0.1866	0.0710	0.2739	0.0310	0.2214	0.1107
PEP_372 (M3)	0.0000	0.0030	0.0000	0.0030	0.0100	0.0099	0.0198	0.0195	-	-	0.0008	0.0030	0.0000	0.0052	0.0034	0.0061
Lac_261 (M0)	1.0000	0.0030	0.8862	0.0033	0.8678	0.0241	0.8361	0.0030	0.8186	0.0058	0.7762	0.0214	0.7457	0.0111	0.6879	0.0431
Lac_262 (M1)	0.0000	0.0030	0.0067	0.0030	0.0138	0.0066	0.0156	0.0077	0.0149	0.0058	0.0168	0.0053	0.0240	0.0091	0.0290	0.0118
Lac_263 (M2)	0.0000	0.0030	0.1071	0.0030	0.1184	0.0307	0.1483	0.0075	0.1666	0.0030	0.2033	0.0138	0.2303	0.0058	0.2831	0.0384
Lac_264 (M3)	0.0000	0.0030	0.0000	0.0030	0.0000	0.0030	0.0000	0.0030	0.0000	0.0030	0.0037	0.0030	0.0000	0.0039	0.0000	0.0071
Lac23_233 (M0)	1.0000	0.0030	0.8975	0.0080	0.8717	0.0279	0.8493	0.0130	0.8312	0.0064	0.7749	0.0238	0.7568	0.0030	0.6916	0.0425
Lac23_234 (M1)	0.0000	0.0030	0.0003	0.0047	0.0065	0.0030	0.0091	0.0030	0.0010	0.0080	0.0097	0.0030	0.0168	0.0030	0.0289	0.0130
Lac23_235 (M2)	0.0000	0.0030	0.1022	0.0033	0.1218	0.0273	0.1417	0.0142	0.1677	0.0030	0.2154	0.0254	0.2264	0.0030	0.2795	0.0295
Ala_260 (M0)	1.0000	0.0030	0.9865	0.0125	0.9771	0.0058	0.9576	0.0171	0.9271	0.0160	0.9015	0.0234	0.8655	0.0181	0.7951	0.0336
Ala_261 (M1)	0.0000	0.0030	0.0000	0.0030	0.0000	0.0030	0.0000	0.0030	0.0000	0.0043	0.0000	0.0068	0.0000	0.0093	0.0000	0.0109
Ala_262 (M2)	0.0000	0.0030	0.0135	0.0125	0.0229	0.0046	0.0424	0.0142	0.0729	0.0115	0.0985	0.0159	0.1299	0.0111	0.2049	0.0491
Ala_263 (M3)	0.0000	0.0030	0.0000	0.0030	0.0000	0.0030	0.0000	0.0030	0.0000	0.0030	0.0000	0.0030	0.0046	0.0030	0.0000	0.0045
Ala23_232 (M0)	1.0000	0.0030	0.9684	0.0306	0.9421	0.0290	0.9248	0.0158	0.9126	0.0030	0.8843	0.0055	0.8455	0.0032	0.7740	0.0566
Ala23_233 (M1)	0.0000	0.0030	0.0000	0.0030	0.0000	0.0030	0.0000	0.0030	0.0021	0.0030	0.0000	0.0067	0.0400	0.0313	0.0062	0.0030
Ala23_234 (M2)	0.0000	0.0030	0.0316	0.0306	0.0579	0.0304	0.0752	0.0185	0.0853	0.0030	0.1157	0.0030	0.1145	0.0280	0.2198	0.0606
Cit_591 (M0)	1.0000	0.0030	0.9474	0.0247	0.9104	0.0141	0.8864	0.0062	0.8555	0.0086	0.8277	0.0084	0.7746	0.0052	0.6575	0.0770
Cit_592 (M1)	0.0000	0.0030	0.0000	0.0030	0.0000	0.0035	0.0000	0.0063	0.0000	0.0110	0.0000	0.0221	0.0000	0.0335	0.0407	0.0030
Cit_593 (M2)	0.0000	0.0030	0.0381	0.0120	0.0775	0.0079	0.1060	0.0091	0.1230	0.0047	0.1460	0.0030	0.1990	0.0230	0.2427	0.0480

Cit_594 (M3)	0.0000	0.0030	0.0120	0.0119	0.0080	0.0078	0.0067	0.0062	0.0214	0.0202	0.0212	0.0178	0.0000	0.0070	0.0154	0.0047
Cit_595 (M4)	0.0000	0.0030	0.0025	0.0030	0.0042	0.0030	0.0002	0.0034	0.0000	0.0054	0.0014	0.0073	0.0114	0.0030	0.0424	0.0250
Cit_596 (M5)	0.0000	0.0030	0.0000	0.0030	0.0000	0.0030	0.0000	0.0030	0.0000	0.0030	0.0038	0.0037	0.0149	0.0145	0.0013	0.0030
Cit_597 (M6)	0.0000	0.0030	0.0000	0.0030	0.0000	0.0030	0.0007	0.0030	0.0000	0.0030	0.0000	0.0030	0.0000	0.0030	0.0000	0.0030
AKG_346 (M0)	1.0000	0.0030	0.9481	0.0516	0.9859	0.0039	0.9754	0.0030	0.9496	0.0093	0.8900	0.0394	0.9037	0.0048	0.8583	0.0144
AKG_347 (M1)	0.0000	0.0030	0.0322	0.0322	0.0000	0.0030	0.0000	0.0030	0.0000	0.0040	0.0008	0.0105	0.0000	0.0197	0.0000	0.0265
AKG_348 (M2)	0.0000	0.0030	0.0146	0.0142	0.0135	0.0039	0.0246	0.0033	0.0371	0.0030	0.0967	0.0415	0.0963	0.0230	0.1295	0.0411
AKG_349 (M3)	0.0000	0.0030	0.0000	0.0030	0.0000	0.0030	0.0000	0.0030	0.0000	0.0030	0.0009	0.0030	0.0000	0.0035	0.0044	0.0030
AKG_350 (M4)	0.0000	0.0030	0.0052	0.0052	0.0006	0.0030	0.0000	0.0030	0.0133	0.0120	0.0115	0.0089	0.0000	0.0046	0.0078	0.0030
AKG_351 (M5)	0.0000	0.0030	0.0000	0.0030	0.0000	0.0030	0.0000	0.0030	0.0000	0.0030	0.0000	0.0030	0.0000	0.0030	0.0000	0.0030
Suc_289 (M0)	1.0000	0.0030	0.9782	0.0216	0.9780	0.0147	0.9996	0.0196	0.9842	0.0226	0.9170	0.0126	0.9259	0.0276	0.8833	0.0102
Suc_290 (M1)	0.0000	0.0030	0.0164	0.0164	0.0000	0.0030	0.0000	0.0030	0.0000	0.0039	0.0316	0.0183	0.0185	0.0054	0.0227	0.0095
Suc_291 (M2)	0.0000	0.0030	0.0000	0.0030	0.0220	0.0152	0.0004	0.0179	0.0122	0.0212	0.0514	0.0030	0.0402	0.0313	0.0940	0.0087
Suc_292 (M3)	0.0000	0.0030	0.0000	0.0030	0.0000	0.0030	0.0000	0.0030	0.0000	0.0030	0.0000	0.0030	0.0000	0.0030	0.0000	0.0047
Suc_293 (M4)	0.0000	0.0030	0.0054	0.0054	0.0000	0.0030	0.0000	0.0030	0.0036	0.0030	0.0000	0.0030	0.0154	0.0122	0.0000	0.0047
Fum_287 (M0)	1.0000	0.0030	0.9542	0.0113	0.9272	0.0174	0.9119	0.0202	0.8914	0.0229	0.8517	0.0303	0.8118	0.0338	0.8175	0.0030
Fum_288 (M1)	0.0000	0.0030	0.0000	0.0030	0.0000	0.0030	0.0000	0.0041	0.0089	0.0030	0.0101	0.0056	0.0236	0.0030	0.0152	0.0175
Fum_289 (M2)	0.0000	0.0030	0.0306	0.0030	0.0661	0.0135	0.0818	0.0183	0.0926	0.0151	0.1306	0.0309	0.1431	0.0194	0.1449	0.0048
Fum_290 (M3)	0.0000	0.0030	0.0093	0.0093	0.0044	0.0044	0.0028	0.0030	0.0071	0.0067	0.0046	0.0031	0.0157	0.0123	0.0129	0.0072
Fum_291 (M4)	0.0000	0.0030	0.0059	0.0059	0.0024	0.0030	0.0035	0.0033	0.0000	0.0030	0.0030	0.0030	0.0058	0.0037	0.0096	0.0066
Mal_419 (M0)	1.0000	0.0030	0.9798	0.0030	0.9482	0.0078	0.9331	0.0075	0.8984	0.0237	0.8525	0.0371	0.8315	0.0221	0.8323	0.0053
Mal_420 (M1)	0.0000	0.0030	0.0021	0.0030	0.0000	0.0030	0.0028	0.0030	0.0000	0.0063	0.0265	0.0117	0.0066	0.0177	0.0235	0.0083
Mal_421 (M2)	0.0000	0.0030	0.0153	0.0061	0.0518	0.0100	0.0641	0.0085	0.0972	0.0264	0.1149	0.0214	0.1551	0.0382	0.1310	0.0030
Mal_422 (M3)	0.0000	0.0030	0.0028	0.0030	0.0000	0.0030	0.0000	0.0030	0.0000	0.0030	0.0003	0.0030	0.0000	0.0033	0.0077	0.0030
Mal_423 (M4)	0.0000	0.0030	0.0000	0.0030	0.0000	0.0030	0.0000	0.0030	0.0044	0.0039	0.0059	0.0048	0.0067	0.0049	0.0056	0.0030
Asp_418 (M0)	1.0000	0.0030	0.9891	0.0030	0.9680	0.0115	0.9634	0.0043	0.9529	0.0030	0.9120	0.0061	0.8907	0.0082	0.8665	0.0076
Asp_419 (M1)	0.0000	0.0030	0.0019	0.0030	0.0058	0.0048	0.0164	0.0146	0.0168	0.0131	0.0142	0.0040	0.0261	0.0068	0.0321	0.0063
Asp_420 (M2)	0.0000	0.0030	0.0090	0.0030	0.0262	0.0067	0.0119	0.0184	0.0259	0.0189	0.0725	0.0030	0.0794	0.0148	0.0924	0.0162

Asp_421 (M3)	0.0000	0.0030	0.0000	0.0030	0.0000	0.0030	0.0065	0.0064	0.0000	0.0030	0.0002	0.0030	0.0038	0.0030	0.0053	0.0030
Asp_422 (M4)	0.0000	0.0030	0.0000	0.0030	0.0000	0.0030	0.0018	0.0030	0.0044	0.0041	0.0012	0.0030	0.0000	0.0030	0.0036	0.0030
Glu_432 (M0)	1.0000	0.0030	0.9997	0.0030	0.9835	0.0062	0.9769	0.0030	0.9462	0.0128	0.8965	0.0329	0.8855	0.0134	0.8305	0.0421
Glu_433 (M1)	0.0000	0.0030	0.0000	0.0030	0.0038	0.0033	0.0036	0.0030	0.0085	0.0046	0.0203	0.0091	0.0136	0.0061	0.0320	0.0055
Glu_434 (M2)	0.0000	0.0030	0.0000	0.0030	0.0111	0.0030	0.0173	0.0039	0.0381	0.0030	0.0717	0.0164	0.0851	0.0118	0.1153	0.0268
Glu_435 (M3)	0.0000	0.0030	0.0000	0.0030	0.0009	0.0030	0.0017	0.0030	0.0033	0.0030	0.0058	0.0042	0.0053	0.0030	0.0109	0.0052
Glu_436 (M4)	0.0000	0.0030	0.0001	0.0030	0.0005	0.0030	0.0005	0.0030	0.0032	0.0030	0.0026	0.0030	0.0062	0.0030	0.0087	0.0030
Glu_437 (M5)	0.0000	0.0030	0.0002	0.0030	0.0000	0.0030	0.0001	0.0030	0.0006	0.0030	0.0031	0.0031	0.0044	0.0043	0.0025	0.0030
Gln_431 (M0)	1.0000	0.0030	1.0000	0.0030	0.9996	0.0030	0.9987	0.0030	0.9971	0.0030	0.9943	0.0057	0.9822	0.0178	0.9976	0.0030
Gln_432 (M1)	0.0000	0.0030	0.0000	0.0030	0.0000	0.0030	0.0000	0.0030	0.0000	0.0030	0.0000	0.0030	0.0120	0.0120	0.0000	0.0030
Gln_433 (M2)	0.0000	0.0030	0.0000	0.0030	0.0000	0.0030	0.0000	0.0030	0.0000	0.0030	0.0000	0.0030	0.0000	0.0030	0.0024	0.0030
Gln_434 (M3)	0.0000	0.0030	0.0000	0.0030	0.0000	0.0030	0.0000	0.0030	0.0018	0.0030	0.0048	0.0048	0.0000	0.0030	0.0000	0.0030
Gln_435 (M4)	0.0000	0.0030	0.0000	0.0030	0.0000	0.0030	0.0000	0.0030	0.0000	0.0030	0.0000	0.0030	0.0000	0.0030	0.0000	0.0030
Gln_436 (M5)	0.0000	0.0030	0.0000	0.0030	0.0004	0.0030	0.0013	0.0030	0.0011	0.0030	0.0009	0.0030	0.0058	0.0058	0.0000	0.0030
Ser_390 (M0)	1.0000	0.0030	0.9931	0.0052	0.9655	0.0140	0.9641	0.0030	0.9631	0.0168	0.9348	0.0072	0.9120	0.0076	0.8705	0.0199
Ser_391 (M1)	0.0000	0.0030	0.0000	0.0030	0.0110	0.0076	0.0152	0.0042	0.0129	0.0080	0.0273	0.0063	0.0416	0.0059	0.0680	0.0125
Ser_392 (M2)	0.0000	0.0030	0.0069	0.0053	0.0235	0.0065	0.0207	0.0065	0.0240	0.0086	0.0380	0.0030	0.0464	0.0030	0.0616	0.0090
Ser_393 (M3)	0.0000	0.0030	0.0000	0.0030	0.0000	0.0030	0.0000	0.0030	0.0000	0.0030	0.0000	0.0030	0.0000	0.0030	0.0000	0.0030
Gly_246 (M0)	1.0000	0.0030	0.9914	0.0085	0.9857	0.0133	0.9824	0.0129	0.9883	0.0030	0.9630	0.0091	0.9519	0.0045	0.9141	0.0186
Gly_247 (M1)	0.0000	0.0030	0.0000	0.0030	0.0124	0.0113	0.0167	0.0121	0.0080	0.0034	0.0370	0.0092	0.0419	0.0101	0.0818	0.0162
Gly_248 (M2)	0.0000	0.0030	0.0086	0.0086	0.0018	0.0030	0.0009	0.0030	0.0038	0.0038	0.0000	0.0030	0.0062	0.0055	0.0042	0.0030

Mass isotopomer distributions were corrected for natural isotope abundance. Errors were calculated as described in the supplementary text.

Table S3 - Measured mass isotopomer distributions and calculated errors from [U-^{13}C]glutamine under growth conditions (mock infection).

**Time (h)**	**0.00**		**0.02**		**0.25**		**0.608**		**1.217**		**3**		**8**		**24**	
3PG_459 (M0)	1.0000	0.0030	1.0000	0.0030	1.0000	0.0030	1.0000	0.0030	1.0000	0.0030	1.0000	0.0030	1.0000	0.0030	1.0000	0.0030
3PG_460 (M1)	0.0000	0.0030	0.0000	0.0030	0.0000	0.0030	0.0000	0.0030	0.0000	0.0030	0.0000	0.0030	0.0000	0.0030	0.0000	0.0030
3PG_461 (M2)	0.0000	0.0030	0.0000	0.0030	0.0000	0.0030	0.0000	0.0030	0.0000	0.0030	0.0000	0.0030	0.0000	0.0030	0.0000	0.0030
3PG_462 (M3)	0.0000	0.0030	0.0000	0.0030	0.0000	0.0030	0.0000	0.0030	0.0000	0.0030	0.0000	0.0030	0.0000	0.0030	0.0000	0.0030
PEP_369 (M0)	1.0000	0.0030	1.0000	0.0030	1.0000	0.0030	1.0000	0.0030	1.0000	0.0030	1.0000	0.0030	1.0000	0.0030	1.0000	0.0030
PEP_370 (M1)	0.0000	0.0030	0.0000	0.0030	0.0000	0.0030	0.0000	0.0030	0.0000	0.0030	0.0000	0.0030	0.0000	0.0030	0.0000	0.0030
PEP_371 (M2)	0.0000	0.0030	0.0000	0.0030	0.0000	0.0030	0.0000	0.0030	0.0000	0.0030	0.0000	0.0030	0.0000	0.0030	0.0000	0.0030
PEP_372 (M3)	0.0000	0.0030	0.0000	0.0030	0.0000	0.0030	0.0000	0.0030	0.0000	0.0030	0.0000	0.0030	0.0000	0.0030	0.0000	0.0030
Lac_261 (M0)	1.0000	0.0030	1.0000	0.0030	1.0000	0.0030	1.0000	0.0037	1.0000	0.0082	0.9990	0.0138	0.9990	0.0173	0.9990	0.0195
Lac_262 (M1)	0.0000	0.0030	0.0000	0.0030	0.0000	0.0030	0.0000	0.0030	0.0000	0.0030	0.0000	0.0030	0.0000	0.0030	0.0000	0.0037
Lac_263 (M2)	0.0000	0.0030	0.0000	0.0030	0.0000	0.0030	0.0000	0.0030	0.0000	0.0030	0.0000	0.0030	0.0000	0.0033	0.0000	0.0038
Lac_264 (M3)	0.0000	0.0030	0.0000	0.0030	0.0000	0.0030	0.0000	0.0033	0.0000	0.0067	0.0014	0.0093	0.0009	0.0111	0.0063	0.0068
Lac23_233 (M0)	1.0000	0.0030	1.0000	0.0030	1.0000	0.0030	1.0000	0.0037	1.0000	0.0082	1.0000	0.0148	1.0000	0.0180	1.0000	0.0202
Lac23_234 (M1)	0.0000	0.0030	0.0000	0.0030	0.0000	0.0030	0.0000	0.0030	0.0000	0.0030	0.0000	0.0030	0.0000	0.0045	0.0000	0.0054
Lac23_235 (M2)	0.0000	0.0030	0.0000	0.0030	0.0000	0.0030	0.0000	0.0037	0.0000	0.0075	0.0000	0.0119	0.0000	0.0135	0.0000	0.0147
Ala_260 (M0)	1.0000	0.0030	1.0000	0.0030	1.0000	0.0030	1.0000	0.0030	1.0000	0.0030	1.0000	0.0081	1.0000	0.0115	1.0000	0.0130
Ala_261 (M1)	0.0000	0.0030	0.0000	0.0030	0.0000	0.0030	0.0000	0.0030	0.0000	0.0030	0.0000	0.0030	0.0000	0.0030	0.0000	0.0030
Ala_262 (M2)	0.0000	0.0030	0.0000	0.0030	0.0000	0.0030	0.0000	0.0030	0.0000	0.0030	0.0000	0.0030	0.0000	0.0030	0.0000	0.0030
Ala_263 (M3)	0.0000	0.0030	0.0000	0.0030	0.0000	0.0030	0.0000	0.0030	0.0000	0.0030	0.0000	0.0061	0.0000	0.0076	0.0000	0.0083
Ala23_232 (M0)	1.0000	0.0030	-	-	-	-	0.9825	0.0167	0.9926	0.0047	0.9620	0.0300	0.9808	0.0078	0.9716	0.0155
Ala23_233 (M1)	0.0000	0.0030	-	-	-	-	0.0000	0.0030	0.0000	0.0030	0.0071	0.0058	0.0000	0.0030	0.0023	0.0030
Ala23_234 (M2)	0.0000	0.0030	-	-	-	-	0.0175	0.0167	0.0074	0.0048	0.0309	0.0241	0.0192	0.0106	0.0261	0.0167
Cit_591 (M0)	1.0000	0.0030	0.9776	0.0221	0.9749	0.0056	0.9330	0.0102	0.8308	0.0117	0.6269	0.0283	0.6201	0.0481	0.5443	0.0250
Cit_592 (M1)	0.0000	0.0030	0.0156	0.0156	0.0000	0.0030	0.0000	0.0030	0.0000	0.0030	0.0000	0.0169	0.0055	0.0307	0.0305	0.0151
Cit_593 (M2)	0.0000	0.0030	0.0000	0.0030	0.0000	0.0030	0.0000	0.0103	0.0002	0.0290	0.0852	0.0163	0.0860	0.0043	0.1131	0.0115

Cit_594 (M3)	0.0000	0.0030	0.0056	0.0056	0.0000	0.0030	0.0226	0.0154	0.0491	0.0290	0.0635	0.0205	0.0737	0.0184	0.0881	0.0246
Cit_595 (M4)	0.0000	0.0030	0.0000	0.0030	0.0000	0.0062	0.0361	0.0047	0.0772	0.0030	0.1310	0.0031	0.1345	0.0175	0.1525	0.0129
Cit_596 (M5)	0.0000	0.0030	0.0000	0.0030	0.0151	0.0050	0.0013	0.0262	0.0428	0.0067	0.0934	0.0189	0.0801	0.0044	0.0715	0.0215
Cit_597 (M6)	0.0000	0.0030	0.0013	0.0030	0.0100	0.0100	0.0069	0.0065	0.0000	0.0030	0.0000	0.0074	0.0000	0.0096	0.0000	0.0115
AKG_346 (M0)	1.0000	0.0030	0.9752	0.0210	0.8610	0.0610	0.8588	0.0279	0.7188	0.0036	0.5101	0.0600	0.5006	0.0167	0.4203	0.0030
AKG_347 (M1)	0.0000	0.0030	0.0000	0.0030	0.0466	0.0466	0.0000	0.0030	0.0000	0.0030	0.0159	0.0062	0.0002	0.0243	0.0154	0.0177
AKG_348 (M2)	0.0000	0.0030	0.0000	0.0030	0.0000	0.0030	0.0000	0.0030	0.0074	0.0030	0.0272	0.0030	0.0374	0.0030	0.0521	0.0062
AKG_349 (M3)	0.0000	0.0030	0.0064	0.0064	0.0000	0.0030	0.0069	0.0030	0.0219	0.0030	0.0856	0.0259	0.1011	0.0177	0.1138	0.0144
AKG_350 (M4)	0.0000	0.0030	0.0000	0.0030	0.0000	0.0030	0.0076	0.0076	0.0106	0.0105	0.0151	0.0138	0.0042	0.0030	0.0020	0.0030
AKG_351 (M5)	0.0000	0.0030	0.0184	0.0147	0.0924	0.0151	0.1267	0.0353	0.2413	0.0072	0.3461	0.0117	0.3566	0.0107	0.3964	0.0032
Suc_289 (M0)	1.0000	0.0030	0.9377	0.0623	0.9182	0.0572	0.8741	0.0289	0.8301	0.0402	0.6306	0.0092	0.6033	0.0714	0.4735	0.0030
Suc_290 (M1)	0.0000	0.0030	0.0000	0.0030	0.0364	0.0364	0.0398	0.0398	0.0434	0.0425	0.0343	0.0225	0.0211	0.0108	0.0407	0.0030
Suc_291 (M2)	0.0000	0.0030	0.0190	0.0190	0.0000	0.0030	0.0240	0.0212	0.0000	0.0170	0.0796	0.0129	0.1072	0.0093	0.1489	0.0330
Suc_292 (M3)	0.0000	0.0030	0.0355	0.0355	0.0000	0.0030	0.0000	0.0030	0.0000	0.0030	0.0000	0.0062	0.0000	0.0094	0.0000	0.0117
Suc_293 (M4)	0.0000	0.0030	0.0078	0.0077	0.0454	0.0212	0.0620	0.0312	0.1265	0.0630	0.2555	0.0383	0.2683	0.0605	0.3369	0.0218
Fum_287 (M0)	1.0000	0.0030	0.9273	0.0727	0.8891	0.0931	0.8983	0.0304	0.8095	0.0338	0.6299	0.0849	0.6183	0.0285	0.5164	0.0847
Fum_288 (M1)	0.0000	0.0030	0.0000	0.0030	0.0000	0.0030	0.0000	0.0030	0.0000	0.0030	0.0282	0.0156	0.0133	0.0158	0.0430	0.0050
Fum_289 (M2)	0.0000	0.0030	0.0586	0.0586	0.0441	0.0438	0.0311	0.0282	0.0457	0.0322	0.0980	0.0492	0.1057	0.0347	0.1183	0.0345
Fum_290 (M3)	0.0000	0.0030	0.0000	0.0030	0.0139	0.0110	0.0132	0.0030	0.0410	0.0160	0.0698	0.0283	0.0773	0.0287	0.1070	0.0529
Fum_291 (M4)	0.0000	0.0030	0.0141	0.0141	0.0529	0.0384	0.0574	0.0030	0.1038	0.0127	0.1741	0.0082	0.1854	0.0191	0.2154	0.0076
Mal_419 (M0)	1.0000	0.0030	0.9640	0.0359	0.9625	0.0227	0.9369	0.0030	0.8155	0.0322	0.6445	0.0647	0.6228	0.0135	0.5204	0.0684
Mal_420 (M1)	0.0000	0.0030	0.0205	0.0205	0.0000	0.0030	0.0000	0.0030	0.0000	0.0030	0.0207	0.0091	0.0135	0.0148	0.0435	0.0062
Mal_421 (M2)	0.0000	0.0030	0.0155	0.0155	0.0030	0.0030	0.0117	0.0095	0.0234	0.0122	0.0844	0.0396	0.0838	0.0165	0.1112	0.0316
Mal_422 (M3)	0.0000	0.0030	0.0000	0.0030	0.0000	0.0042	0.0037	0.0139	0.0492	0.0099	0.0859	0.0180	0.0917	0.0118	0.1037	0.0149
Mal_423 (M4)	0.0000	0.0030	0.0000	0.0030	0.0345	0.0242	0.0477	0.0030	0.1119	0.0115	0.1644	0.0030	0.1882	0.0030	0.2212	0.0158
Asp_418 (M0)	1.0000	0.0030	0.9919	0.0081	0.9467	0.0471	0.9616	0.0084	0.9220	0.0048	0.7343	0.0614	0.6964	0.0136	0.6665	0.0030
Asp_419 (M1)	0.0000	0.0030	0.0000	0.0030	0.0287	0.0287	0.0012	0.0030	0.0000	0.0030	0.0001	0.0070	0.0155	0.0068	0.0423	0.0122
Asp_420 (M2)	0.0000	0.0030	0.0000	0.0030	0.0086	0.0085	0.0000	0.0030	0.0097	0.0039	0.0826	0.0529	0.0684	0.0143	0.0705	0.0055

Asp_421 (M3)	0.0000	0.0030	0.0081	0.0081	0.0000	0.0030	0.0049	0.0030	0.0152	0.0050	0.0595	0.0139	0.0625	0.0030	0.0644	0.0031
Asp_422 (M4)	0.0000	0.0030	0.0000	0.0030	0.0160	0.0116	0.0324	0.0110	0.0531	0.0031	0.1235	0.0030	0.1573	0.0038	0.1564	0.0120
Glu_432 (M0)	1.0000	0.0030	0.9641	0.0321	0.8741	0.0480	0.8560	0.0251	0.7087	0.0135	0.5101	0.0598	0.4743	0.0094	0.4058	0.0117
Glu_433 (M1)	0.0000	0.0030	0.0132	0.0132	0.0042	0.0042	0.0012	0.0030	0.0032	0.0030	0.0184	0.0087	0.0239	0.0030	0.0399	0.0070
Glu_434 (M2)	0.0000	0.0030	0.0000	0.0030	0.0000	0.0030	0.0000	0.0030	0.0075	0.0030	0.0317	0.0067	0.0376	0.0030	0.0496	0.0037
Glu_435 (M3)	0.0000	0.0030	0.0000	0.0030	0.0012	0.0030	0.0043	0.0030	0.0296	0.0093	0.0855	0.0259	0.0920	0.0087	0.1095	0.0102
Glu_436 (M4)	0.0000	0.0030	0.0014	0.0030	0.0041	0.0041	0.0059	0.0059	0.0097	0.0096	0.0123	0.0110	0.0152	0.0120	0.0141	0.0096
Glu_437 (M5)	0.0000	0.0030	0.0214	0.0175	0.1164	0.0390	0.1326	0.0296	0.2413	0.0073	0.3420	0.0076	0.3569	0.0103	0.3811	0.0187
Gln_431 (M0)	1.0000	0.0030	0.3004	0.0125	0.2048	0.0421	0.2102	0.0367	0.2186	0.0282	0.1977	0.0491	0.2227	0.0242	0.2034	0.0434
Gln_432 (M1)	0.0000	0.0030	0.0000	0.0030	0.0014	0.0030	0.0000	0.0030	0.0000	0.0030	0.0021	0.0030	0.0000	0.0030	0.0046	0.0046
Gln_433 (M2)	0.0000	0.0030	0.0152	0.0152	0.0053	0.0053	0.0000	0.0030	0.0000	0.0030	0.0020	0.0030	0.0000	0.0030	0.0000	0.0030
Gln_434 (M3)	0.0000	0.0030	0.0056	0.0056	0.0076	0.0076	0.0038	0.0038	0.0056	0.0056	0.0092	0.0092	0.0050	0.0050	0.0086	0.0086
Gln_435 (M4)	0.0000	0.0030	0.0270	0.0270	0.0345	0.0345	0.0267	0.0267	0.0317	0.0317	0.0336	0.0336	0.0303	0.0303	0.0358	0.0358
Gln_436 (M5)	0.0000	0.0030	0.6518	0.0354	0.7465	0.0067	0.7594	0.0062	0.7441	0.0091	0.7553	0.0030	0.7421	0.0112	0.7475	0.0056
Ser_390 (M0)	1.0000	0.0030	1.0000	0.0030	1.0000	0.0030	1.0000	0.0030	1.0000	0.0030	1.0000	0.0030	1.0000	0.0030	1.0000	0.0030
Ser_391 (M1)	0.0000	0.0030	0.0000	0.0030	0.0000	0.0030	0.0000	0.0030	0.0000	0.0030	0.0000	0.0030	0.0000	0.0030	0.0000	0.0030
Ser_392 (M2)	0.0000	0.0030	0.0000	0.0030	0.0000	0.0030	0.0000	0.0030	0.0000	0.0030	0.0000	0.0030	0.0000	0.0030	0.0000	0.0030
Ser_393 (M3)	0.0000	0.0030	0.0000	0.0030	0.0000	0.0030	0.0000	0.0030	0.0000	0.0030	0.0000	0.0030	0.0000	0.0030	0.0000	0.0030
Gly_246 (M0)	1.0000	0.0030	1.0000	0.0030	1.0000	0.0030	1.0000	0.0030	1.0000	0.0030	1.0000	0.0030	1.0000	0.0030	1.0000	0.0030
Gly_247 (M1)	0.0000	0.0030	0.0000	0.0030	0.0000	0.0030	0.0000	0.0030	0.0000	0.0030	0.0000	0.0030	0.0000	0.0030	0.0000	0.0030
Gly_248 (M2)	0.0000	0.0030	0.0000	0.0030	0.0000	0.0030	0.0000	0.0030	0.0000	0.0030	0.0000	0.0030	0.0000	0.0030	0.0000	0.0030

Mass isotopomer distributions were corrected for natural isotope abundance. Errors were calculated as described in the supplementary text.

Table S4 - Measured mass isotopomer distributions and calculated errors from [1,2-^{13}C]glucose during CAV2 infection.

**Time (h)**	**0.00**		**0.02**		**0.25**		**0.608**		**1.217**		**3**		**8**		**24**	
3PG_459 (M0)	1.0000	0.0030	0.6798	0.0975	0.7059	0.0620	-	-	-	-	0.6800	0.0145	0.6089	0.0411	0.6849	0.0478
3PG_460 (M1)	0.0000	0.0030	0.0734	0.0624	0.0000	0.0117	-	-	-	-	0.0353	0.0182	0.0417	0.0214	0.0079	0.0135
3PG_461 (M2)	0.0000	0.0030	0.2159	0.0043	0.2729	0.0528	-	-	-	-	0.2668	0.0182	0.3136	0.0084	0.3072	0.0239
3PG_462 (M3)	0.0000	0.0030	0.0309	0.0309	0.0211	0.0209	-	-	-	-	0.0179	0.0143	0.0358	0.0280	0.0000	0.0103
PEP_369 (M0)	1.0000	0.0030	0.7389	0.0385	0.7080	0.0600	-	-	-	-	0.6644	0.0300	0.6438	0.0061	0.6455	0.0084
PEP_370 (M1)	0.0000	0.0030	0.0602	0.0491	0.0380	0.0262	-	-	-	-	0.0381	0.0210	0.0231	0.0030	0.0118	0.0097
PEP_371 (M2)	0.0000	0.0030	0.1890	0.0227	0.2540	0.0340	-	-	-	-	0.2831	0.0030	0.2818	0.0402	0.3291	0.0030
PEP_372 (M3)	0.0000	0.0030	0.0120	0.0120	0.0000	0.0030	-	-	-	-	0.0144	0.0109	0.0512	0.0434	0.0136	0.0033
Lac_261 (M0)	1.0000	0.0030	0.8722	0.0030	0.8393	0.0046	0.8359	0.0030	0.8448	0.0245	0.7716	0.0204	0.7393	0.0216	0.6790	0.0721
Lac_262 (M1)	0.0000	0.0030	0.0043	0.0030	0.0209	0.0127	0.0198	0.0107	0.0108	0.0030	0.0179	0.0048	0.0159	0.0030	0.0295	0.0121
Lac_263 (M2)	0.0000	0.0030	0.1235	0.0030	0.1326	0.0151	0.1443	0.0119	0.1444	0.0238	0.2105	0.0180	0.2449	0.0270	0.2915	0.0672
Lac_264 (M3)	0.0000	0.0030	0.0000	0.0030	0.0071	0.0069	0.0000	0.0030	0.0000	0.0030	0.0000	0.0030	0.0000	0.0054	0.0000	0.0071
Lac23_233 (M0)	1.0000	0.0030	0.8703	0.0030	0.8710	0.0270	0.8490	0.0144	0.8553	0.0341	0.7715	0.0221	0.7482	0.0152	0.6894	0.0648
Lac23_234 (M1)	0.0000	0.0030	0.0070	0.0030	0.0176	0.0094	0.0062	0.0030	0.0154	0.0051	0.0184	0.0059	0.0159	0.0030	0.0274	0.0111
Lac23_235 (M2)	0.0000	0.0030	0.1226	0.0030	0.1114	0.0364	0.1448	0.0114	0.1293	0.0392	0.2100	0.0162	0.2359	0.0143	0.2832	0.0538
Ala_260 (M0)	1.0000	0.0030	1.0000	0.0129	0.8665	0.0469	0.9391	0.0323	0.9234	0.0246	0.8948	0.0123	0.8812	0.0165	0.7887	0.0704
Ala_261 (M1)	0.0000	0.0030	0.0000	0.0030	0.0300	0.0255	0.0000	0.0051	0.0000	0.0059	0.0000	0.0074	0.0000	0.0091	0.0000	0.0099
Ala_262 (M2)	0.0000	0.0030	0.0000	0.0123	0.0855	0.0034	0.0609	0.0269	0.0766	0.0181	0.1052	0.0035	0.1188	0.0045	0.2113	0.0843
Ala_263 (M3)	0.0000	0.0030	0.0000	0.0030	0.0180	0.0179	0.0000	0.0030	0.0000	0.0030	0.0000	0.0030	0.0000	0.0031	0.0000	0.0041
Ala23_232 (M0)	1.0000	0.0030	0.9666	0.0204	0.8503	0.0631	0.9299	0.0228	0.9329	0.0336	0.8858	0.0030	0.8416	0.0245	0.7685	0.0924
Ala23_233 (M1)	0.0000	0.0030	0.0000	0.0030	0.0549	0.0504	0.0000	0.0051	0.0000	0.0058	0.0000	0.0071	0.0063	0.0030	0.0087	0.0030
Ala23_234 (M2)	0.0000	0.0030	0.0334	0.0212	0.0949	0.0128	0.0701	0.0177	0.0671	0.0279	0.1142	0.0047	0.1521	0.0266	0.2228	0.0928
Cit_591 (M0)	1.0000	0.0030	0.9686	0.0245	0.9267	0.0169	0.8790	0.0207	0.8553	0.0030	0.7910	0.0112	0.7496	0.0030	0.6764	0.0464
Cit_592 (M1)	0.0000	0.0030	0.0000	0.0030	0.0000	0.0036	0.0000	0.0075	0.0000	0.0132	0.0083	0.0162	0.0244	0.0116	0.0210	0.0244
Cit_593 (M2)	0.0000	0.0030	0.0087	0.0030	0.0498	0.0030	0.1053	0.0171	0.1364	0.0153	0.1440	0.0137	0.1987	0.0122	0.2404	0.0416

Cit_594 (M3)	0.0000	0.0030	0.0200	0.0200	0.0011	0.0030	0.0000	0.0030	0.0000	0.0030	0.0277	0.0232	0.0020	0.0069	0.0354	0.0229
Cit_595 (M4)	0.0000	0.0030	0.0000	0.0030	0.0027	0.0030	0.0139	0.0102	0.0000	0.0062	0.0290	0.0181	0.0215	0.0052	0.0199	0.0030
Cit_596 (M5)	0.0000	0.0030	0.0000	0.0030	0.0197	0.0197	0.0000	0.0030	0.0000	0.0030	0.0000	0.0030	0.0009	0.0030	0.0011	0.0030
Cit_597 (M6)	0.0000	0.0030	0.0027	0.0030	0.0000	0.0030	0.0017	0.0030	0.0083	0.0082	0.0000	0.0030	0.0028	0.0030	0.0057	0.0053
AKG_346 (M0)	1.0000	0.0030	0.9981	0.0030	0.9807	0.0038	0.9762	0.0059	0.9406	0.0113	0.9143	0.0065	0.8944	0.0079	0.8610	0.0030
AKG_347 (M1)	0.0000	0.0030	0.0000	0.0030	0.0000	0.0030	0.0000	0.0030	0.0000	0.0060	0.0000	0.0130	0.0000	0.0216	0.0000	0.0299
AKG_348 (M2)	0.0000	0.0030	0.0019	0.0030	0.0000	0.0137	0.0238	0.0030	0.0446	0.0051	0.0788	0.0181	0.0960	0.0147	0.1255	0.0288
AKG_349 (M3)	0.0000	0.0030	0.0000	0.0030	0.0000	0.0030	0.0000	0.0030	0.0005	0.0030	0.0069	0.0047	0.0027	0.0030	0.0046	0.0030
AKG_350 (M4)	0.0000	0.0030	0.0000	0.0030	0.0084	0.0079	0.0000	0.0030	0.0143	0.0126	0.0000	0.0033	0.0069	0.0030	0.0089	0.0030
AKG_351 (M5)	0.0000	0.0030	0.0000	0.0030	0.0109	0.0109	0.0000	0.0030	0.0000	0.0030	0.0000	0.0030	0.0000	0.0030	0.0000	0.0030
Suc_289 (M0)	1.0000	0.0030	1.0000	0.0094	0.9670	0.0062	-	-	0.9523	0.0095	0.9337	0.0202	0.8970	0.0150	0.8943	0.0368
Suc_290 (M1)	0.0000	0.0030	0.0000	0.0030	0.0112	0.0091	-	-	0.0170	0.0092	0.0015	0.0146	0.0243	0.0030	0.0239	0.0114
Suc_291 (M2)	0.0000	0.0030	0.0000	0.0089	0.0218	0.0030	-	-	0.0275	0.0198	0.0584	0.0075	0.0778	0.0063	0.0819	0.0144
Suc_292 (M3)	0.0000	0.0030	0.0000	0.0030	0.0000	0.0030	-	-	0.0000	0.0030	0.0000	0.0030	0.0000	0.0041	0.0000	0.0061
Suc_293 (M4)	0.0000	0.0030	0.0000	0.0030	0.0000	0.0030	-	-	0.0032	0.0030	0.0064	0.0041	0.0009	0.0030	0.0000	0.0049
Fum_287 (M0)	1.0000	0.0030	0.9627	0.0088	0.8952	0.0458	0.9031	0.0239	0.8711	0.0377	0.8424	0.0351	0.8321	0.0115	0.8202	0.0030
Fum_288 (M1)	0.0000	0.0030	0.0000	0.0030	0.0000	0.0040	0.0000	0.0062	0.0071	0.0030	0.0175	0.0030	0.0112	0.0157	0.0249	0.0101
Fum_289 (M2)	0.0000	0.0030	0.0287	0.0030	0.0993	0.0446	0.0894	0.0234	0.0930	0.0136	0.1271	0.0262	0.1448	0.0226	0.1374	0.0040
Fum_290 (M3)	0.0000	0.0030	0.0086	0.0086	0.0056	0.0055	0.0075	0.0071	0.0227	0.0219	0.0029	0.0030	0.0044	0.0030	0.0092	0.0030
Fum_291 (M4)	0.0000	0.0030	0.0000	0.0030	0.0000	0.0030	0.0000	0.0030	0.0060	0.0052	0.0100	0.0085	0.0075	0.0049	0.0083	0.0049
Mal_419 (M0)	1.0000	0.0030	0.9879	0.0030	0.8931	0.0628	0.9389	0.0030	0.8527	0.0676	0.8462	0.0414	0.8118	0.0420	0.8378	0.0060
Mal_420 (M1)	0.0000	0.0030	0.0000	0.0030	0.0492	0.0463	0.0000	0.0050	0.0580	0.0496	0.0406	0.0242	0.0563	0.0306	0.0276	0.0064
Mal_421 (M2)	0.0000	0.0030	0.0086	0.0030	0.0345	0.0064	0.0611	0.0066	0.0893	0.0195	0.1101	0.0176	0.1199	0.0061	0.1243	0.0030
Mal_422 (M3)	0.0000	0.0030	0.0035	0.0035	0.0121	0.0120	0.0000	0.0030	0.0000	0.0030	0.0004	0.0030	0.0041	0.0030	0.0036	0.0030
Mal_423 (M4)	0.0000	0.0030	0.0000	0.0030	0.0111	0.0109	0.0000	0.0030	0.0000	0.0030	0.0026	0.0030	0.0078	0.0054	0.0067	0.0036
Asp_418 (M0)	1.0000	0.0030	0.9862	0.0030	0.9621	0.0104	0.9513	0.0096	0.9644	0.0194	0.8965	0.0184	0.9008	0.0200	0.9063	0.0460
Asp_419 (M1)	0.0000	0.0030	0.0000	0.0030	0.0000	0.0030	0.0000	0.0032	0.0000	0.0057	0.0190	0.0069	0.0258	0.0050	0.0124	0.0157
Asp_420 (M2)	0.0000	0.0030	0.0138	0.0034	0.0379	0.0125	0.0487	0.0132	0.0304	0.0178	0.0845	0.0139	0.0705	0.0226	0.0756	0.0283

Asp_421 (M3)	0.0000	0.0030	0.0000	0.0030	0.0000	0.0030	0.0000	0.0030	0.0052	0.0047	0.0000	0.0030	0.0000	0.0035	0.0052	0.0030
Asp_422 (M4)	0.0000	0.0030	0.0000	0.0030	0.0000	0.0030	0.0000	0.0030	0.0000	0.0030	0.0000	0.0030	0.0029	0.0030	0.0005	0.0030
Glu_432 (M0)	1.0000	0.0030	0.9980	0.0030	0.9669	0.0228	0.9726	0.0032	0.9458	0.0119	0.9014	0.0256	0.8723	0.0211	0.8361	0.0289
Glu_433 (M1)	0.0000	0.0030	0.0011	0.0030	0.0161	0.0153	0.0071	0.0048	0.0027	0.0030	0.0174	0.0055	0.0259	0.0057	0.0324	0.0039
Glu_434 (M2)	0.0000	0.0030	0.0000	0.0030	0.0139	0.0048	0.0161	0.0048	0.0459	0.0109	0.0695	0.0134	0.0839	0.0073	0.1130	0.0209
Glu_435 (M3)	0.0000	0.0030	0.0000	0.0030	0.0000	0.0030	0.0018	0.0030	0.0011	0.0030	0.0032	0.0030	0.0064	0.0030	0.0080	0.0030
Glu_436 (M4)	0.0000	0.0030	0.0009	0.0030	0.0014	0.0030	0.0010	0.0030	0.0019	0.0030	0.0068	0.0037	0.0089	0.0036	0.0080	0.0030
Glu_437 (M5)	0.0000	0.0030	0.0000	0.0030	0.0016	0.0030	0.0014	0.0030	0.0026	0.0030	0.0018	0.0030	0.0027	0.0030	0.0025	0.0030
Gln_431 (M0)	1.0000	0.0030	0.9924	0.0076	0.9932	0.0068	0.9906	0.0094	0.9951	0.0049	1.0000	0.0030	0.9922	0.0078	0.9948	0.0052
Gln_432 (M1)	0.0000	0.0030	0.0000	0.0030	0.0000	0.0030	0.0000	0.0030	0.0000	0.0030	0.0000	0.0030	0.0000	0.0030	0.0000	0.0030
Gln_433 (M2)	0.0000	0.0030	0.0000	0.0030	0.0000	0.0030	0.0000	0.0030	0.0000	0.0030	0.0000	0.0030	0.0000	0.0030	0.0024	0.0030
Gln_434 (M3)	0.0000	0.0030	0.0030	0.0030	0.0000	0.0030	0.0010	0.0030	0.0000	0.0030	0.0000	0.0030	0.0000	0.0030	0.0000	0.0030
Gln_435 (M4)	0.0000	0.0030	0.0000	0.0030	0.0006	0.0030	0.0000	0.0030	0.0000	0.0030	0.0000	0.0030	0.0018	0.0030	0.0000	0.0030
Gln_436 (M5)	0.0000	0.0030	0.0046	0.0046	0.0062	0.0062	0.0084	0.0084	0.0049	0.0049	0.0000	0.0030	0.0061	0.0061	0.0028	0.0030
Ser_390 (M0)	1.0000	0.0030	0.9870	0.0038	0.9437	0.0091	0.9440	0.0040	0.9681	0.0252	0.9328	0.0030	0.9231	0.0107	0.8560	0.0328
Ser_391 (M1)	0.0000	0.0030	0.0061	0.0055	0.0247	0.0074	0.0262	0.0062	0.0113	0.0112	0.0308	0.0030	0.0267	0.0143	0.0762	0.0144
Ser_392 (M2)	0.0000	0.0030	0.0070	0.0030	0.0315	0.0030	0.0293	0.0030	0.0206	0.0137	0.0364	0.0032	0.0502	0.0047	0.0678	0.0198
Ser_393 (M3)	0.0000	0.0030	0.0000	0.0030	0.0002	0.0030	0.0005	0.0030	0.0000	0.0030	0.0000	0.0030	0.0000	0.0030	0.0000	0.0030
Gly_246 (M0)	1.0000	0.0030	0.9980	0.0030	0.9610	0.0097	0.9770	0.0092	0.9529	0.0123	0.9730	0.0132	0.9502	0.0034	0.9215	0.0303
Gly_247 (M1)	0.0000	0.0030	0.0000	0.0030	0.0390	0.0097	0.0230	0.0091	0.0407	0.0061	0.0256	0.0142	0.0429	0.0030	0.0761	0.0294
Gly_248 (M2)	0.0000	0.0030	0.0020	0.0030	0.0000	0.0030	0.0000	0.0030	0.0064	0.0062	0.0014	0.0030	0.0069	0.0058	0.0024	0.0030

Mass isotopomer distributions were corrected for natural isotope abundance. Errors were calculated as described in the supplementary text.

Table S5 - Measured mass isotopomer distributions and calculated errors from [U-^{13}C]glutamine during CAV2 infection.

Time (h)	0.00		0.02		0.25		0.608		1.217		3		8		24	
3PG_459 (M0)	1.0000	0.0030	1.0000	0.0030	1.0000	0.0030	1.0000	0.0030	1.0000	0.0030	1.0000	0.0030	1.0000	0.0030	1.0000	0.0030
3PG_460 (M1)	0.0000	0.0030	0.0000	0.0030	0.0000	0.0030	0.0000	0.0030	0.0000	0.0030	0.0000	0.0030	0.0000	0.0030	0.0000	0.0030
3PG_461 (M2)	0.0000	0.0030	0.0000	0.0030	0.0000	0.0030	0.0000	0.0030	0.0000	0.0030	0.0000	0.0030	0.0000	0.0030	0.0000	0.0030
3PG_462 (M3)	0.0000	0.0030	0.0000	0.0030	0.0000	0.0030	0.0000	0.0030	0.0000	0.0030	0.0000	0.0030	0.0000	0.0030	0.0000	0.0030
PEP_369 (M0)	1.0000	0.0030	1.0000	0.0030	1.0000	0.0030	1.0000	0.0030	1.0000	0.0030	1.0000	0.0030	1.0000	0.0030	1.0000	0.0030
PEP_370 (M1)	0.0000	0.0030	0.0000	0.0030	0.0000	0.0030	0.0000	0.0030	0.0000	0.0030	0.0000	0.0030	0.0000	0.0030	0.0000	0.0030
PEP_371 (M2)	0.0000	0.0030	0.0000	0.0030	0.0000	0.0030	0.0000	0.0030	0.0000	0.0030	0.0000	0.0030	0.0000	0.0030	0.0000	0.0030
PEP_372 (M3)	0.0000	0.0030	0.0000	0.0030	0.0000	0.0030	0.0000	0.0030	0.0000	0.0030	0.0000	0.0030	0.0000	0.0030	0.0000	0.0030
Lac_261 (M0)	1.0000	0.0030	1.0000	0.0030	1.0000	0.0030	1.0000	0.0051	1.0000	0.0088	1.0000	0.0140	0.9990	0.0169	0.9990	0.0204
Lac_262 (M1)	0.0000	0.0030	0.0000	0.0030	0.0000	0.0030	0.0000	0.0030	0.0000	0.0030	0.0000	0.0030	0.0000	0.0030	0.0000	0.0039
Lac_263 (M2)	0.0000	0.0030	0.0000	0.0030	0.0000	0.0030	0.0000	0.0030	0.0000	0.0030	0.0000	0.0030	0.0000	0.0032	0.0000	0.0040
Lac_264 (M3)	0.0000	0.0030	0.0000	0.0030	0.0000	0.0030	0.0000	0.0043	0.0000	0.0069	0.0000	0.0100	0.0040	0.0078	0.0079	0.0057
Lac23_233 (M0)	1.0000	0.0030	1.0000	0.0030	1.0000	0.0030	1.0000	0.0052	1.0000	0.0088	0.9996	0.0134	0.9978	0.0154	0.9911	0.0121
Lac23_234 (M1)	0.0000	0.0030	0.0000	0.0030	0.0000	0.0030	0.0000	0.0030	0.0000	0.0030	0.0000	0.0030	0.0000	0.0043	0.0000	0.0057
Lac23_235 (M2)	0.0000	0.0030	0.0000	0.0030	0.0000	0.0030	0.0000	0.0047	0.0000	0.0076	0.0004	0.0107	0.0022	0.0111	0.0089	0.0063
Ala_260 (M0)	1.0000	0.0030	1.0000	0.0030	1.0000	0.0030	1.0000	0.0030	1.0000	0.0049	1.0000	0.0079	1.0000	0.0101	1.0000	0.0122
Ala_261 (M1)	0.0000	0.0030	0.0000	0.0030	0.0000	0.0030	0.0000	0.0030	0.0000	0.0030	0.0000	0.0030	0.0000	0.0030	0.0000	0.0030
Ala_262 (M2)	0.0000	0.0030	0.0000	0.0030	0.0000	0.0030	0.0000	0.0030	0.0000	0.0030	0.0000	0.0030	0.0000	0.0030	0.0000	0.0030
Ala_263 (M3)	0.0000	0.0030	0.0000	0.0030	0.0000	0.0030	0.0000	0.0030	0.0000	0.0039	0.0000	0.0057	0.0000	0.0067	0.0000	0.0077
Ala23_232 (M0)	1.0000	0.0030	0.9928	0.0072	0.9688	0.0303	0.9754	0.0219	0.9876	0.0075	0.9674	0.0248	0.9608	0.0293	0.9729	0.0151
Ala23_233 (M1)	0.0000	0.0030	0.0000	0.0030	0.0000	0.0030	0.0039	0.0037	0.0000	0.0030	0.0131	0.0115	0.0086	0.0062	0.0000	0.0032
Ala23_234 (M2)	0.0000	0.0030	0.0072	0.0072	0.0312	0.0303	0.0207	0.0182	0.0124	0.0082	0.0195	0.0133	0.0305	0.0230	0.0271	0.0184
Cit_591 (M0)	1.0000	0.0030	0.9888	0.0109	0.9527	0.0270	0.9369	0.0105	0.8049	0.0277	0.7233	0.0448	0.5757	0.0030	0.4839	0.0067
Cit_592 (M1)	0.0000	0.0030	0.0000	0.0030	0.0000	0.0030	0.0000	0.0030	0.0000	0.0045	0.0000	0.0171	0.0058	0.0276	0.0530	0.0057
Cit_593 (M2)	0.0000	0.0030	0.0000	0.0030	0.0164	0.0134	0.0000	0.0118	0.0123	0.0171	0.0552	0.0077	0.0912	0.0051	0.1133	0.0100

Cit_594 (M3)	0.0000	0.0030	0.0112	0.0112	0.0000	0.0030	0.0110	0.0048	0.0574	0.0407	0.0634	0.0252	0.0990	0.0443	0.1151	0.0461
Cit_595 (M4)	0.0000	0.0030	0.0000	0.0030	0.0205	0.0115	0.0455	0.0143	0.0682	0.0030	0.0708	0.0433	0.1438	0.0053	0.1578	0.0030
Cit_596 (M5)	0.0000	0.0030	0.0000	0.0030	0.0104	0.0038	0.0000	0.0226	0.0573	0.0093	0.0872	0.0045	0.0845	0.0177	0.0770	0.0421
Cit_597 (M6)	0.0000	0.0030	0.0000	0.0030	0.0000	0.0030	0.0065	0.0059	0.0000	0.0030	0.0000	0.0063	0.0000	0.0094	0.0000	0.0125
AKG_346 (M0)	1.0000	0.0030	0.9896	0.0067	0.8713	0.0581	0.8307	0.0148	0.6572	0.0902	0.5267	0.0858	0.4897	0.0178	0.3809	0.0255
AKG_347 (M1)	0.0000	0.0030	0.0000	0.0030	0.0000	0.0030	0.0000	0.0030	0.0000	0.0030	0.0064	0.0044	0.0006	0.0223	0.0267	0.0087
AKG_348 (M2)	0.0000	0.0030	0.0000	0.0030	0.0000	0.0030	0.0272	0.0235	0.0210	0.0113	0.0724	0.0487	0.0500	0.0130	0.0489	0.0030
AKG_349 (M3)	0.0000	0.0030	0.0062	0.0061	0.0098	0.0070	0.0081	0.0030	0.0387	0.0142	0.0800	0.0257	0.0948	0.0148	0.1194	0.0148
AKG_350 (M4)	0.0000	0.0030	0.0042	0.0042	0.0044	0.0044	0.0000	0.0030	0.0014	0.0030	0.0030	0.0030	0.0074	0.0044	0.0137	0.0082
AKG_351 (M5)	0.0000	0.0030	0.0000	0.0036	0.1145	0.0476	0.1340	0.0060	0.2817	0.0661	0.3115	0.0141	0.3575	0.0078	0.4104	0.0115
Suc_289 (M0)	1.0000	0.0030	0.9981	0.0030	0.8971	0.0436	0.9343	0.0649	0.8227	0.0382	0.6982	0.0311	0.6446	0.0676	0.4764	0.0144
Suc_290 (M1)	0.0000	0.0030	0.0000	0.0030	0.0488	0.0486	0.0000	0.0030	0.0000	0.0038	0.0251	0.0108	0.0149	0.0141	0.0405	0.0034
Suc_291 (M2)	0.0000	0.0030	0.0000	0.0030	0.0000	0.0030	0.0000	0.0113	0.0503	0.0228	0.0313	0.0298	0.0873	0.0030	0.1588	0.0447
Suc_292 (M3)	0.0000	0.0030	0.0000	0.0030	0.0000	0.0030	0.0000	0.0036	0.0000	0.0065	0.0013	0.0100	0.0017	0.0138	0.0027	0.0174
Suc_293 (M4)	0.0000	0.0030	0.0019	0.0030	0.0540	0.0030	0.0657	0.0491	0.1270	0.0506	0.2442	0.0030	0.2516	0.0382	0.3215	0.0094
Fum_287 (M0)	1.0000	0.0030	0.9623	0.0358	0.9094	0.0467	0.9201	0.0206	0.8032	0.0256	0.7361	0.0077	0.6030	0.0497	0.4856	0.0960
Fum_288 (M1)	0.0000	0.0030	0.0000	0.0030	0.0210	0.0208	0.0000	0.0030	0.0000	0.0040	0.0000	0.0137	0.0203	0.0064	0.0401	0.0030
Fum_289 (M2)	0.0000	0.0030	0.0278	0.0278	0.0186	0.0162	0.0282	0.0195	0.0467	0.0254	0.0877	0.0402	0.1002	0.0308	0.1216	0.0323
Fum_290 (M3)	0.0000	0.0030	0.0088	0.0086	0.0180	0.0129	0.0059	0.0067	0.0388	0.0158	0.0175	0.0196	0.0919	0.0452	0.1105	0.0548
Fum_291 (M4)	0.0000	0.0030	0.0012	0.0030	0.0330	0.0032	0.0458	0.0321	0.1112	0.0117	0.1586	0.0145	0.1847	0.0199	0.2422	0.0085
Mal_419 (M0)	1.0000	0.0030	0.9856	0.0137	0.9346	0.0333	0.9206	0.0049	0.7998	0.0409	0.6601	0.0653	0.5963	0.0459	0.4973	0.0702
Mal_420 (M1)	0.0000	0.0030	0.0072	0.0072	0.0301	0.0299	0.0000	0.0030	0.0099	0.0067	0.0185	0.0060	0.0302	0.0045	0.0308	0.0081
Mal_421 (M2)	0.0000	0.0030	0.0072	0.0072	0.0052	0.0036	0.0304	0.0238	0.0273	0.0100	0.0930	0.0503	0.0869	0.0224	0.1191	0.0353
Mal_422 (M3)	0.0000	0.0030	0.0000	0.0030	0.0000	0.0051	0.0000	0.0168	0.0411	0.0051	0.0827	0.0174	0.0890	0.0057	0.1265	0.0275
Mal_423 (M4)	0.0000	0.0030	0.0000	0.0030	0.0301	0.0049	0.0491	0.0109	0.1219	0.0192	0.1456	0.0086	0.1976	0.0133	0.2263	0.0155
Asp_418 (M0)	1.0000	0.0030	0.9979	0.0030	0.9491	0.0310	0.9568	0.0103	0.8880	0.0056	0.7464	0.0488	0.6838	0.0240	0.5851	0.0558
Asp_419 (M1)	0.0000	0.0030	0.0000	0.0030	0.0157	0.0156	0.0000	0.0030	0.0000	0.0030	0.0574	0.0486	0.0476	0.0269	0.0551	0.0230
Asp_420 (M2)	0.0000	0.0030	0.0000	0.0030	0.0084	0.0074	0.0201	0.0160	0.0200	0.0086	0.0557	0.0247	0.0538	0.0030	0.0802	0.0109

Asp_421 (M3)	0.0000	0.0030	0.0000	0.0030	0.0032	0.0030	0.0000	0.0107	0.0196	0.0045	0.0370	0.0120	0.0700	0.0030	0.0966	0.0136
Asp_422 (M4)	0.0000	0.0030	0.0021	0.0030	0.0236	0.0079	0.0232	0.0150	0.0725	0.0036	0.1035	0.0124	0.1448	0.0058	0.1830	0.0084
Glu_432 (M0)	1.0000	0.0030	0.9902	0.0061	0.8756	0.0530	0.8601	0.0157	0.6929	0.0527	0.5460	0.0640	0.5460	0.0419	0.4522	0.0502
Glu_433 (M1)	0.0000	0.0030	0.0000	0.0030	0.0014	0.0030	0.0013	0.0030	0.0000	0.0030	0.0196	0.0099	0.0196	0.0030	0.0317	0.0030
Glu_434 (M2)	0.0000	0.0030	0.0000	0.0030	0.0000	0.0030	0.0000	0.0030	0.0092	0.0030	0.0321	0.0105	0.0321	0.0030	0.0345	0.0124
Glu_435 (M3)	0.0000	0.0030	0.0003	0.0030	0.0037	0.0030	0.0024	0.0051	0.0334	0.0127	0.0781	0.0284	0.0781	0.0030	0.0967	0.0030
Glu_436 (M4)	0.0000	0.0030	0.0018	0.0030	0.0062	0.0062	0.0064	0.0064	0.0118	0.0116	0.0151	0.0141	0.0151	0.0122	0.0153	0.0102
Glu_437 (M5)	0.0000	0.0030	0.0078	0.0039	0.1131	0.0438	0.1299	0.0151	0.2527	0.0294	0.3092	0.0030	0.3092	0.0527	0.3696	0.0432
Gln_431 (M0)	1.0000	0.0030	0.2581	0.0087	0.2152	0.0220	0.2282	0.0091	0.2073	0.0300	0.1982	0.0391	0.2130	0.0243	0.2020	0.0354
Gln_432 (M1)	0.0000	0.0030	0.0000	0.0030	0.0000	0.0030	0.0000	0.0030	0.0000	0.0030	0.0081	0.0081	0.0000	0.0030	0.0006	0.0030
Gln_433 (M2)	0.0000	0.0030	0.0000	0.0030	0.0033	0.0033	0.0000	0.0030	0.0000	0.0030	0.0000	0.0030	0.0000	0.0030	0.0073	0.0073
Gln_434 (M3)	0.0000	0.0030	0.0034	0.0034	0.0033	0.0033	0.0038	0.0038	0.0025	0.0030	0.0033	0.0033	0.0053	0.0053	0.0076	0.0076
Gln_435 (M4)	0.0000	0.0030	0.0311	0.0311	0.0310	0.0310	0.0302	0.0302	0.0352	0.0352	0.0330	0.0330	0.0348	0.0348	0.0349	0.0349
Gln_436 (M5)	0.0000	0.0030	0.7074	0.0260	0.7471	0.0155	0.7377	0.0250	0.7550	0.0076	0.7573	0.0054	0.7469	0.0158	0.7476	0.0151
Ser_390 (M0)	1.0000	0.0030	1.0000	0.0030	1.0000	0.0030	1.0000	0.0030	1.0000	0.0030	1.0000	0.0030	1.0000	0.0030	1.0000	0.0030
Ser_391 (M1)	0.0000	0.0030	0.0000	0.0030	0.0000	0.0030	0.0000	0.0030	0.0000	0.0030	0.0000	0.0030	0.0000	0.0030	0.0000	0.0030
Ser_392 (M2)	0.0000	0.0030	0.0000	0.0030	0.0000	0.0030	0.0000	0.0030	0.0000	0.0030	0.0000	0.0030	0.0000	0.0030	0.0000	0.0030
Ser_393 (M3)	0.0000	0.0030	0.0000	0.0030	0.0000	0.0030	0.0000	0.0030	0.0000	0.0030	0.0000	0.0030	0.0000	0.0030	0.0000	0.0030
Gly_246 (M0)	1.0000	0.0030	1.0000	0.0030	1.0000	0.0030	1.0000	0.0030	1.0000	0.0030	1.0000	0.0030	1.0000	0.0030	1.0000	0.0030
Gly_247 (M1)	0.0000	0.0030	0.0000	0.0030	0.0000	0.0030	0.0000	0.0030	0.0000	0.0030	0.0000	0.0030	0.0000	0.0030	0.0000	0.0030
Gly_248 (M2)	0.0000	0.0030	0.0000	0.0030	0.0000	0.0030	0.0000	0.0030	0.0000	0.0030	0.0000	0.0030	0.0000	0.0030	0.0000	0.0030

Mass isotopomer distributions were corrected for natural isotope abundance. Errors were calculated as described in the supplementary text.

Table S6 - Estimated fluxes and 95% confidence intervals from combined [1,2-^{13}C]glucose and [U-^{13}C]glutamine experiments.

			**Growth (mock infection)**			**CAV2 infection**		
**Reaction**			**Flux**	**LB**	**UB**	**Flux**	**LB**	**UB**
G6P <-> F6P	R1	net	601.34	588.77	608.03	750.40	737.99	763.82
G6P <-> F6P		exch.	0.00	0	Inf	2409.70	0	Inf
F6P -> FBP	R2		618.84	614.82	620.80	779.12	775.13	783.89
FBP <-> DHAP + GAP	R3	net	618.84	614.82	620.80	779.12	775.13	783.89
FBP <-> DHAP + GAP		exch.	1197.40	0	Inf	92.76	0	Inf
DHAP <-> GAP	R4	net	618.10	614.23	620.24	777.53	773.24	782.31
DHAP <-> GAP		exch.	5.18E+05	0	Inf	2590.80	0	Inf
GAP <-> 3PG	R5	net	1245.70	1241.60	1248.00	1571.00	1566.10	1574.70
GAP <-> 3PG		exch.	1.42E+04	0	Inf	0.00	0	Inf
3PG <-> PEP	R6	net	1237.40	1199.30	1244.00	1563.90	1537.20	1571.30
3PG <-> PEP		exch.	954.92	0	Inf	1739.90	0	Inf
PEP -> Pyr.c	R7		1237.40	1199.30	1244.00	1563.90	1537.20	1571.30
G6P -> P5P + CO2	R8		27.64	21.81	39.64	46.10	32.10	56.63
P5P + P5P <-> GAP + S7P	R9	net	8.75	6.70	13.72	14.36	9.72	17.93
P5P + P5P <-> GAP + S7P		exch.	0.86	0	11.19	3.31	0	15.91
S7P + GAP <-> E4P + F6P	R10	net	8.75	6.70	13.72	14.36	9.72	17.93
S7P + GAP <-> E4P + F6P		exch.	389.84	335.71	507.24	380.08	310.17	466.19
E4P + P5P <-> GAP + F6P	R11	net	8.75	6.70	13.72	14.36	9.72	17.93
E4P + P5P <-> GAP + F6P		exch.	461.72	171.47	Inf	1.53E+05	84.08	Inf
Pyr.c <-> Lac	R12	net	1244.80	1228.80	1258.20	1564.80	1547.80	1581.60
Pyr.c <-> Lac		exch.	0.00	0	280.77	0.00	0	130.20
Pyr.c <-> Ala	R13	net	35.65	28.00	43.54	45.60	36.19	53.11
Pyr.c <-> Ala		exch.	0.00	0	84.19	0.00	0	61.26
Pyr.m -> AcCoA.m + CO2	R14		40.67	36.52	45.51	71.36	64.06	77.61
Pyr.m + CO2 -> OAA	R15		112.22	105.99	123.07	133.77	118.76	148.57
OAA + AcCoA.m -> Cit	R16		77.56	74.49	80.67	112.87	107.59	116.91
Cit <-> AKG + CO2	R17	net	62.37	59.10	66.14	80.13	74.72	84.85
Cit <-> AKG + CO2		exch.	20.40	17.10	23.41	41.18	34.62	47.19
AKG -> SucCoA + CO2	R18		133.96	128.29	138.53	177.38	169.19	185.34
SucCoA <-> Suc	R19	net	133.96	128.29	138.53	177.38	169.19	185.34
SucCoA <-> Suc		exch.	8704.90	0	Inf	531.62	0	Inf
Suc <-> Fum	R20	net	139.46	133.90	144.54	191.02	183.01	200.06
Suc <-> Fum		exch.	21.55	7.75	38.98	78.36	53.77	106.39
Fum <-> Mal	R21	net	140.27	134.69	146.90	192.09	183.55	202.06
Fum <-> Mal		exch.	8.80E+04	2612.00	Inf	4.52E+05	5882.90	Inf
Mal <-> OAA	R22	net	-40.63	-53.92	-30.21	-44.62	-59.70	-28.85
Mal <-> OAA		exch.	2545.50	1244.10	Inf	6790.80	1616.30	Inf
Mal -> Pyr.m + CO2	R23		180.90	167.16	195.62	236.72	219.38	257.64

Cit -> OAA + AcCoA.c	R24		15.18	12.07	18.84	32.73	28.51	36.45
Gln -> Glu	R25		80.69	80.22	81.08	112.96	112.49	113.50
AKG <-> Glu	R26	net	-71.58	-76.09	-67.39	-97.25	-103.74	-91.45
AKG <-> Glu		exch.	1.43E+05	5233.10	Inf	2872.90	1281.20	NaN
Asn <-> Asp	R27	net	-3.49	-7.13	-0.19	-1.88	-7.16	0.13
Asn <-> Asp		exch.	15.07	5.48	36.36	6.74	3.96	20.60
Asp <-> OAA	R28	net	-9.21	-13.07	-5.56	-9.01	-14.10	-6.26
Asp <-> OAA		exch.	0.00	0	12.99	0.00	0	17.26
3PG -> Ser	R29		8.26	2.82	57.00	7.06	4.03	36.07
Ser -> Pyr.c	R30		14.97	5.46	58.18	14.86	10.40	47.15
Ser <-> Gly + C1	R31	net	1.16	0.54	1.86	2.54	1.12	3.50
Ser <-> Gly + C1		exch.	40.35	7.16	361.54	17.40	9.64	81.27
Glu <-> Pro	R32	net	-2.22	-5.68	0.84	0.48	-3.78	4.20
Glu <-> Pro		exch.	32.94	24.15	47.97	98.45	66.91	159.60
Val breakdown	R33		0.00	0	2.83	6.17	NaN	8.92
Ile breakdown	R34		5.50	4.37	6.23	6.89	6.15	7.69
Leu breakdown	R35		6.94	6.06	7.84	7.92	5.99	9.55
Thr breakdown	R36		4.03	3.30	4.75	4.35	3.20	5.54
Phe -> Tyr	R37		0.31	0	0.80	0.00	0	0.91
Tyr breakdown	R38		0.82	0.00	1.45	1.07	0.66	2.13
Met breakdown	R39		0.00	0	0.37	0.58	0	1.74
Lys breakdown	R40		2.45	0.60	4.34	2.19	NaN	3.38
His -> Glu + C1	R41		0.28	0	0.85	0.00	0	0.48
Arg -> Glu + Urea.snk	R42		3.95	2.09	61.61	7.16	5.42	9.60
Glu + CO2 -> Arg	R43		0.00	0	3.45	0.00	0	12.25
Pyr.c <-> Pyr.m	R44	net	-28.01	-34.17	-16.68	-31.58	-43.66	-22.01
Pyr.c <-> Pyr.m		exch.	189.06	169.86	216.11	274.99	250.75	305.19
CO2 <-> CO2.ext	R45	net	339.05	327.33	356.52	489.54	458.55	513.30
CO2 <-> CO2.ext		exch.	0.00	0	Inf	2342.40	0	Inf
Glc.ext -> G6P	R46		630.77	630.77	630.77	800.34	800.34	800.34
Lac <-> Lac.ext	R47	net	1244.80	1228.80	1258.20	1564.80	1547.80	1581.60
Lac <-> Lac.ext		exch.	399.76	280.50	467.49	665.82	548.37	812.43
Ala <-> Ala.ext	R48	net	32.03	24.32	39.78	37.81	30.17	45.90
Ala <-> Ala.ext		exch.	40.64	29.91	151.39	71.13	58.47	149.67
Gln.ext -> Gln	R49		82.63	82.63	82.63	117.14	117.14	117.14
Glu <-> Glu.ext	R50	net	13.23	10.78	15.52	17.38	13.04	22.68
Glu <-> Glu.ext		exch.	0.00	0	2.26	0.00	0	2.06
Asp <-> Asp.ext	R51	net	2.88	1.69	4.07	1.01	-0.59	2.68
Asp <-> Asp.ext		exch.	0.23	0	3.41	0.00	0	3.46
Asn <-> Asn.ext	R52	net	1.75	-1.61	5.40	-1.86	-3.87	3.80
Asn <-> Asn.ext		exch.	1.50	0	3.72	0.00	0	8.01
Ser.ext <-> Ser	R53	net	11.09	6.48	14.64	17.83	14.98	20.91
Ser.ext <-> Ser		exch.	0.00	0	185.03	11.88	0	133.74
Gly <-> Gly.ext	R54	net	1.23	0.55	2.13	-1.65	-2.89	-0.38

Gly <-> Gly.ext		exch.	12.51	0	169.53	0.00	0	24.39
Pro.ext <-> Pro	R55	net	4.11	1.11	7.79	3.58	-0.57	8.26
Pro.ext <-> Pro		exch.	0.00	0	3.51	0.00	0	NaN
Val.ext -> Val	R56		2.50	2.06	5.00	11.57	8.70	14.88
Ile.ext -> Ile	R57		7.45	6.62	8.12	11.10	10.51	11.74
Leu.ext -> Leu	R58		10.34	9.57	11.17	15.24	12.76	17.03
Thr.ext -> Thr	R59		6.35	5.47	7.34	9.36	8.07	10.69
Phe.ext -> Phe	R60		1.63	1.25	1.96	2.84	2.51	3.81
Tyr.ext -> Tyr	R61		1.60	1.24	1.95	3.43	3.17	3.71
Met.ext -> Met	R62		0.83	0.68	1.16	2.37	1.72	3.52
Lys.ext -> Lys	R63		5.88	3.89	7.53	9.58	8.72	10.35
His.ext -> His	R64		1.15	0.73	1.58	1.86	1.62	2.31
Arg.ext <-> Arg	R65	net	6.22	4.41	8.51	12.05	10.72	13.47
Arg.ext <-> Arg		exch.	166.37	0	Inf	0.00	0	Inf
Biomass formation	R66		0.0111	0.0089	0.0138	0.02	0.02	0.03

Estimated fluxes and 95% confidence intervals are in units of nmol/10^6 cells/h. LB – lower bound; UB – upper bound.
